# Medroxyprogesterone Acetate (MPA) Enhances HIV-1 Accumulation and Release in Primary Cervical Epithelial Cells by Inhibiting Lysosomal Activity

**DOI:** 10.3390/pathogens10091192

**Published:** 2021-09-14

**Authors:** Xiangxu Jia, Qiujia Shao, Ahsen R. Chaudhry, Ballington L. Kinlock, Michael G. Izban, Hong-Ying Zhang, Fernando Villalta, James E. K. Hildreth, Bindong Liu

**Affiliations:** 1 Center for AIDS Health Disparities Research, Meharry Medical College, Nashville, TN 37208, USA; xjia@mmc.edu (X.J.); qshao@mmc.edu (Q.S.); bkinlock@coppin.edu (B.L.K.); fvillalta@mmc.edu (F.V.); jhildreth@mmc.edu (J.E.K.H.); 2 Department of Obstetrics and Gynecology, Meharry Medical College, Nashville, TN 37208, USA; achaudhry@mmc.edu; 3 Department of Pathology, Meharry Medical College, Nashville, TN 37208, USA; mizban@mmc.edu; 4 Nanjing Municipal Center of Disease Control and Prevention, Nanjing 210003, China; xiao99now@aliyun.com; 5 Department of Microbiology, Immunology and Physiology, Meharry Medical College, Nashville, TN 37208, USA

**Keywords:** HIV-1, Medroxyprogesterone acetate, MPA, transmission, lysosome activity

## Abstract

Medroxyprogesterone acetate (MPA) is one of the most widely used contraceptives in the world. Epidemiologic studies have uncovered a possible link between the use of MPA and an increased risk of HIV-1 transmission. However, the understanding of the mechanism is still limited. Our previous publication demonstrated that the lysosomal activity in human vaginal epithelial cells attenuated the trafficking of viral particles during HIV-1 transcytosis. In this study, we show that treating human primary cervical epithelial cells with MPA led to a reduction in lysosomal activity. This reduction caused an increase in the intracellular HIV-1 accumulation and, consequently, an increase in viral release. Our study uncovers a novel mechanism by which MPA enhances HIV-1 release in primary cervical epithelial cells, thus providing vital information for HIV intervention and prevention.

## 1. Introduction

HIV-1 is transmitted predominantly through mucosal epithelial exposure [1]. Approximately 40% of new HIV-1 infections originate from exposure in the female genital tract [2]. During sexual intercourse, HIV-1 transmission from male to female is more efficient than from female to male. As a result, the lower female reproductive tract has become the predominant site for HIV-1 entry in most new HIV-1 infections worldwide. This also means that HIV-1 infection disproportionately affects women [3]. 

The use of medroxyprogesterone acetate (MPA) is one of the most popular contraception methods in areas of high HIV seroprevalence. Interestingly, MPA is also one of the most widely used contraceptives in the world, especially in areas endemic for HIV-1 [4]. Epidemiologic reports have uncovered a possible link between the use of MPA and an increased risk of HIV-1 acquisition and transmission. This link has been shown by numerous groups and succinctly summarized [4,5,6,7]. More recent meta-analysis of high-quality studies showed a significant risk of HIV acquisition associated with MPA use [8,9,10]. However, because a recent open-label randomized Evidence for Contraceptive Options and HIV Outcomes (ECHO) trial did not find a substantial difference in HIV risk among the use of MPA, a copper intrauterine device (IUD), and a levonorgestrel (LNG) implant among African women, the association between MPA use and increased HIV risk has become controversial [11,12,13].

Studies using animal models have corroborated the findings of epidemiological studies that identified the link between MPA use and higher HIV-1 acquisition. For instance, macaques that were given subcutaneous MPA implants had a higher rate of contracting SIV than those that were given a placebo [14]. The link between physiological elevation of progesterone level and viral susceptibility was also apparent in studies where macaques were challenged with viral doses during their undisturbed menstrual cycles. Specifically, virus transmission occurred most frequently during the high-progesterone, late luteal phase of their menstrual cycle, underscoring the critical role of hormonal regulation during HIV-1 mucosal transmission [15]. 

Several mechanisms have been proposed to explain why MPA may lead to enhanced HIV-1 transmission. Both endogenous and exogenous progesterone have been shown to thin or impair the integrity of the cervicovaginal mucosa, consequently enhancing HIV acquisition [14,16,17,18,19,20]. MPA use has also been reported to elevate levels of inflammatory mediators that activate HIV targets cells and promote their recruitment to the cervicovaginal mucosa [17,21,22,23,24,25,26], suppress innate or adaptive immunity against HIV [27,28], modulate the composition of vaginal microbiota [29], and enhance transcytosis of HIV [30], among other effects. However, further studies are warranted to comprehensively understand the action of MPA on HIV transmission.

Several studies using cervical, intestinal, and other epithelial cell types show that HIV-1 traffics through an intact epithelial cell layer via transcytosis [31,32,33,34,35]. Using confocal microscopy and 3D surface reconstruction, findings from Maher et al. demonstrated that HIV-1 binds and penetrates the external surface of cervicovaginal epithelial cells [36]. This critical finding supports the idea that HIV-1 can cross the vaginal epithelial layer through either transcytosis or tight junctions between the epithelial cells. Carias et al. also reported that vaginal epithelial cells may play a bigger role in HIV-1 acquisition than previously believed and argued that the virus is passing between the cells through a diffusive percolation mechanism, penetrating through areas where junctions are absent [37]. In our previous publication, we defined the negative effect of lysosomal activity on HIV-1 transcytosis [38]. Here, we show that MPA treatment of human primary cervical/vaginal epithelial cells led to a reduction in lysosomal activity, consequently resulting in increased intracellular HIV-1 accumulation and virus release with implications of higher viral transmission rates. These data suggest a novel mechanism by which MPA influences HIV susceptibility. 

## 2. Results

### 2.1. MPA Treatment Enhances Intracellular HIV-1 Accumulation

**Figure 1:**
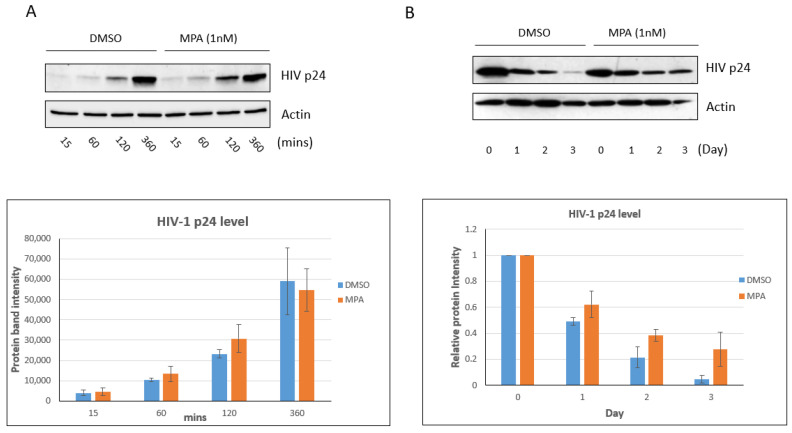
MPA enhances intracellular HIV accumulation in VK2/E6E7 cells. (**A**) VK2 cells were treated with 1 nM MPA for 1 h. Control cells were treated with DMSO alone. After the 1 h treatment, the cells were exposed to 60 ng p24 content of HIV-1 IIIB virus for 15, 60, 120, or 360 min, respectively. After trypsinization to remove viral particles attached to the cell surface, the samples were analyzed by Western blot. The bar graph shows the densitometry analysis of the Western blot result. The results shown are representative of three independent experiments. (**B**) VK2 cells were treated with 1 nM MPA for 1 h. Control cells were treated with DMSO alone. After the 1 h treatment, the cells were exposed to 60 ng p24 content of HIV-1 IIIB virus for 3 h. The viral particles on the cell surface were removed by trypsinization and PBS wash. The cells were then cultured in fresh medium containing 1 nM MPA for the indicated days. The Day 0 samples were harvested immediately after the addition of fresh medium. The harvested samples were analyzed by Western blot. The bar graph shows the densitometry analysis of the Western blot result. Day 0 was set as 1. The value of Days 1, 2 and 3 represent their ratio to Day 0. The results shown are representative of three independent experiments.

It has been shown that MPA can enhance HIV-1 uptake and transcytosis [30]. In our previous publication, we established a method for measuring HIV accumulation in human vaginal epithelial cell line VK2/E6E7 cells (termed VK2 thereafter) using Western blot [38]. Here, we used the same method to investigate the influence of MPA on HIV accumulation in VK2 cells. We treated VK2 cells with 1 nM MPA, a physiologically relevant concentration [39,40], for 1 h. Then, we exposed the cells to 60 ng p24 protein content of HIV-1 IIIB virus for 15, 60, 120, and 360 min. After trypsinization and wash, we analyzed the samples by Western blot. As shown in Figure 1A, MPA pretreatment did not significantly affect HIV virion uptake/accumulation in VK2 cells within the 6 h timeframe. Conversely, exposure to MPA for a longer period markedly changed the level of intracellular HIV. As shown in Figure 1B, the levels of virus remained significantly elevated within the cells after a three-day incubation period. Therefore, in our experimental system, MPA treatment can modulate intracellular viral accumulation upon prolonged exposure. Despite these findings, our results show the efficiency in viral uptake seemed to be limited in VK2 cells. In our system, about 0.1% of input HIV-1 IIIB virus was up taken by the VK2 cells in a 6-hour incubation (Figure S1).

Next, we wanted to determine if the same can occur in primary cervical epithelial cells. To do this, we examined primary cervical epithelial cells from 12 different donors. 

**Figure 2:**
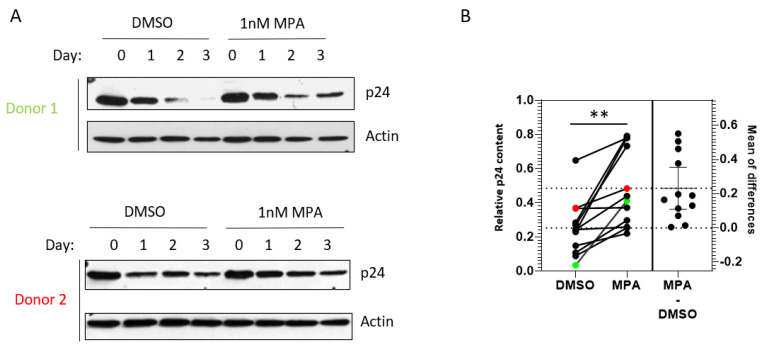
MPA enhances intracellular HIV-1 accumulation in primary cervical epithelial cells. (**A**) Primary cervical epithelial cells from 12 donors were treated with 1 nM MPA for 1 h. Control cells were treated with DMSO alone. After the 1 h treatment, the cells were exposed to HIV-1 IIIB and processed as described in Figure 1B. Representative data from two donor samples are shown. (**B**) A chart depicting the densitometry analysis of p24 accumulation, indicative of HIV-1 accumulation, in primary cervical epithelial cell samples from 12 different donors as described in Figure 2A. The relative p24 content (Y-axis) represents the ratio of p24 level on Day 3 over that on Day 0 (*p* = 0.0016, paired *t*-test). The data from Donors 1 and 2 in Figure 2A are labeled as green and red dots, respectively.

Figure 2A shows representative p24 Western blots of samples from two donors. We observed that cells treated with MPA showed significantly higher HIV accumulation, as indicated by higher p24 accumulation, three days after infection, compared to untreated cells (Figure 2B; *p* = 0.0016, paired *t*-test). Our data from primary cells support the notion that MPA-induced enhancement of viral accumulation is physiologically relevant. 

### 2.2. MPA Reduces Lysosomal Activity

**Figure 3:**
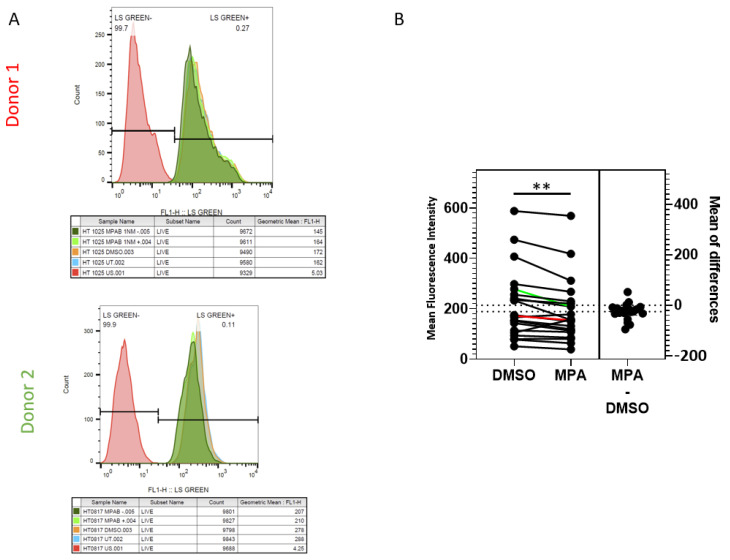
MPA reduces lysosomal activity as revealed by LysoSensorTM staining. (**A**) Primary cervical epithelial cells from 21 donors were pretreated as described in the methods section. After two PBS washes, the samples were stained with 0.5 µM LysoSensorTM Green DND-189 for 15 min at 37 °C. After two PBS washes, the cells were analyzed using BD FACSCalibur. Representative data from two donor samples are shown. (**B**) A chart depicting the analysis of LysoSensorTM staining of primary cervical epithelial cell samples from 21 donors as described in Figure 3A (*p* = 0.0016, paired *t*-test). The Y-axis shows the mean fluorescence intensity of each sample. The results of Donors 1 and 2 in Figure 3A were labeled in red and green lines, respectively.

In our previous study, we found that the lysosomal degradation pathway was involved in HIV trafficking in VK2 cells [38]. When we used a lysosomal inhibitor cocktail of pepstatin A, leupeptin, and E-64 to block the lysosomal degradation pathway, higher levels of HIV accumulated in VK2 cells. Here, we postulate that MPA inhibits lysosomal activity, consequently enhancing intracellular HIV accumulation. To determine whether MPA directly affects lysosomal activity in primary cervical epithelial cells, we treated these cells with 1 nM MPA and assessed lysosomal activity using LysoSensor^TM^ by measuring changes in mean fluorescent intensity (MFI). Figure 3A depicts representative flow cytometry data of cells from two donors. Our examination of primary cervical epithelial cells from 21 donors showed that MPA treatment significantly reduced the LysoSensor fluorescence staining intensity in all samples compared to their DMSO-treated controls (Figure 3B; *p* = 0.0016, paired *t*-test). Even though the reduction in fluorescence intensity seemed marginal, these minor changes may represent a dramatic shift in pH value due to the pH indicator feature of LysoSensor. Such pH shifts can have a big influence on lysosomal activity [41]. These results indicate that MPA treatment reduced lysosomal activity in these cells. 

**Figure 4:**
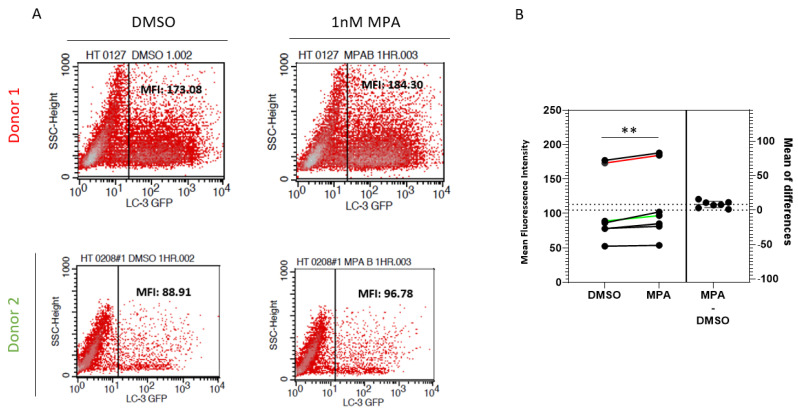
MPA enhances the stability of the LC-3 protein. (**A**) pEX-GFP-hLC3WT, an LC3-GFP fusion protein expression plasmid, was transfected into primary cervical epithelial cell samples from seven donors. The transfected cells were treated with 1 nM MPA as described in the methods section. Control cells were treated with DMSO alone. GFP intensity was measured by flow cytometry using BD FACSCalibur^TM^. (**B**) A chart depicting the analysis of GFP intensity of samples from seven donors as described in Figure 4A. The Y-axis shows the mean fluorescence intensity of each sample. The result of Donors 1 and 2 was labeled as red and green, respectively.

Lysosomal activity has been shown to inversely affect the intracellular human microtubule-associated protein 1 light chain 3 (LC3) pool [42]. Therefore, to test the lysosomal activity functionally, we evaluated the effect of MPA on the stability of LC3 in primary cervical epithelial cells. To do this, we tested the effect of MPA on LC3 levels using pEX-GFP-hLC3WT, an expression plasmid for the LC3-GFP fusion protein. First, we transfected primary cervical epithelial cells from seven donors with pEX-GFP-hLC3WT. Then, we treated these cells with MPA and measured their fluorescence by flow cytometry. Figure 4A depicts representative flow cytometry data of cells from two donors. The MFI of the GFP-positive cells increased significantly after MPA treatment for all seven primary cell samples examined, indicative of higher LC3 levels, compared to their DMSO-treated controls (Figure 4B; *p* = 0.0049, paired *t*-test). Tanida et al. showed that the lysosome slightly degraded the LC3 protein under normal conditions, with degradation becoming more extensive under starvation conditions [43]. These findings are consistent with our observations in Figure 4. Taken together, these findings further support the notion that MPA reduces lysosomal activity.

### 2.3. MPA Enhances HIV-1 Release from Human Primary Cervical Epithelial Cells 

**Figure 5:**
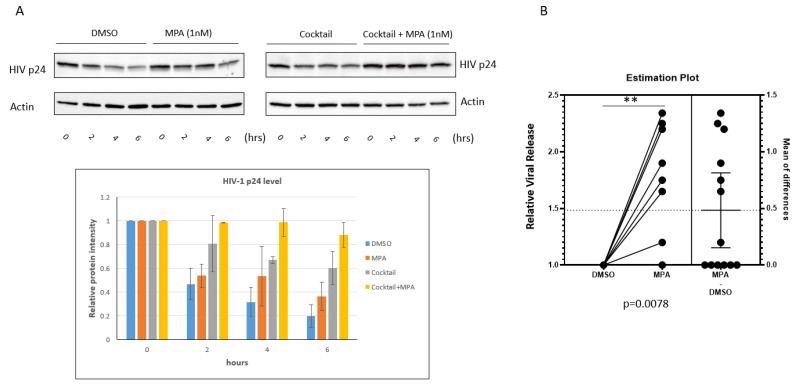
MPA enhances HIV-1 release from primary cervical epithelial cells. (**A**) DMSO, MPA, lysosomal inhibitor cocktail, or the combination of MPA and lysosomal inhibitor cocktail treated VK2 cells were exposed to the CH040 virus for 3 h. Trypsin (0.03%) was used to treat the cells at room temperature for 1 min to remove viral particles attached to the cell surface. Cells were cultured for the indicated periods of time. After the incubation, cells were harvested and subjected to Western blot analysis. The MPA treatment experiment (left panel of Figure 5A) was performed three times independently. The cocktail treatment experiment (right panel of Figure 5A) was independently performed twice. The bar graph shows the densitometry analysis of the Western blot result. (**B**) A box and whisker plot showing relative viral release from human primary cervical epithelial cells pretreated with 1 nM MPA for 1 h. Control samples were treated with DMSO alone. The pretreated cells were exposed to HIV-1 CH040 for 3 h. Then, the cells were treated with a low concentration of trypsin (0.03%) at room temperature. After a thorough wash, the cells were cultured for 4 h in fresh medium containing either 1 nM MPA or DMSO. Finally, the viral samples were harvested and analyzed by qRT-PCR to measure viral release. Samples from 13 donors were analyzed together as one group (*p* = 0.0109, paired *t*-test). The value for the DMSO samples was set as 1.

Most HIV-1 transmission events worldwide result from heterosexual sex with an infected partner. Based on analyses of viral complexity in the blood during the first several weeks of infection, approximately 80% of heterosexual transmission events and infections are established from a single HIV-1 variant known as the transmitted/founder virus (T/F virus) (Reviewed [44]). To test if MPA-induced enhancement of HIV-1 is physiologically relevant, we used human primary cervical epithelial cells and transmitted/founder virus HIV-1 CH040. First, we examined the accumulation of CH040 in VK2 cells. Surprisingly, the turnaround of HIV-1 CH040 in VK2 cells was dramatically faster than that of HIV IIIB. As shown in Figure 5A, the level of HIV p24 dramatically decreased at the 6 h time point. Both MPA and the cocktail of lysosomal inhibitors conferred enhancement of HIV-1 p24 accumulation at the 4 and 6 h time point. Interestingly, the combination of MPA and the cocktail of lysosomal inhibitors showed additive effects on the enhancement of p24 accumulation with almost 100% effectiveness. This finding sheds light on the future investigation of the molecular mechanism of MPA regulating lysosome activity. Finally, we tested the effect of MPA on viral release from the human primary cervical cells. Human primary cervical cells from 13 donors were treated with either 1 nM MPA or DMSO only. Then, we exposed the treated cells to HIV-1 CH040 for 3 h. After treatment with low-concentration trypsin and PBS wash, we cultured the cells for 4 h in fresh medium containing either 1 nM MPA dissolved in DMSO or DMSO only. Then, we harvested the viral samples to measure viral release using qRT-PCR. As shown in Figure 5B, MPA treatment significantly enhanced viral release compared to treatment with DMSO alone (*p* = 0.0078, paired *t*-test) even though viral released from six donors kept unchanged. Our data suggest that MPA enhances viral release from human primary cervical epithelial cells.

## 3. Discussion

The safety of contraception is an important public health issue. Contraception benefits women directly by allowing them to control their reproductive health and reduce the number of unintended pregnancies. It also has several indirect benefits, such as reducing the number of abortions, decreasing maternal and infant morbidity and mortality, and lowering the risk of vertical HIV-1 transmission. MPA is the most commonly used injectable contraceptive. While MPA is highly effective and long-lasting, there have been concerns regarding the association of MPA use with higher incidence of HIV-1 transmission. However, a recent large open-label randomized ECHO trial did not find a significant difference in HIV risk among the use of MPA, a copper IUD, and an LNG implant among African women [11]. Based on the result of this ECHO trial, the WHO took quick action to change the Medical Eligibility Criteria for MPA use among women at high risk for HIV infection from category 2 to category 1; the latter recommends the use of MPA in any circumstances. This action drew criticism due to concerns with the ECHO trial in trial design and data interpretation [12,13,45,46,47]. As MPA is widely used in areas endemic for HIV-1, it is critical to understand how MPA influences HIV-1 transmission so that appropriate measures can be instituted to prevent HIV-1 transmission. 

In our previous study, we established a cell-based model to study the transcytosis of HIV in VK2 cells [38]. In that study, we found the transcytosis of HIV-1 in vaginal epithelial cells to be inversely related to lysosomal activity. Here, we used this same model to investigate whether MPA can influence HIV transcytosis in primary cervical epithelial cells. Indeed, we found that MPA enhanced both intracellular HIV-1 accumulation and extracellular viral release. Interestingly, our data also showed that MPA inhibited lysosomal activity of these cells. Taken together, our findings suggest that MPA enhances HIV intracellular accumulation and extracellular release by inhibiting lysosomal activity. Several studies have revealed the association of lysosomal activity with HIV-1 pathogenesis [48,49,50,51,52,53,54,55,56,57,58,59]. While these reports agree that inhibiting lysosomal activity would increase HIV-1 infectivity, whether lysosomal activity would affect viral release remains controversial. In this regard, Fredericksen et al. showed that lysosomotropic agents do not alter HIV-1 release [57].

In contrast, Schaeffer et al. showed that inhibition of lysosomal activity increases the extracellular release of HIV-1 [48]. Our findings are consistent with those of Schaeffer et al. in that we observed an increase in viral accumulation and release when we inhibited the lysosomal activity of HIV-infected cells. It is well accepted that lysosomes use their hydrolytic enzymes to destroy pathogens that enter the cell. Logically, for epithelial cells that exhibit strong endocytosis activity, it is not surprising that viral accumulation and release increase when lysosomal activity is inhibited. 

At this time, we need to interpret our data cautiously due to the use of an *ex vivo* assay, a small sample size, and factors that are beyond our control, such as menstruation status. Additionally, it is worth mentioning that MPA treatment induces various immunological changes in the female genital tract [60]. The altered immune landscape might also provide a possible explanation for the increased HIV-1 p24 level in the MPA-treated cells. This interesting hypothesis warrants further investigation. Nonetheless, our study uncovers a plausible explanation as to how MPA can enhance HIV transmission. Importantly, it provides vital preliminary evidence to policy experts responsible for disseminating information about the link between progesterone use in contraception and HIV-1 acquisition/transmission.

## 4. Materials and Methods

### 4.1. Cell Lines, HIV-1 Virus, Plasmids, Reagents, and Antibodies

Human vaginal epithelial cell line VK2/E6E7 cells (ATCC CRL-2616) were cultured in defined keratinocyte serum-free medium (SFM) (Thermo Fisher Scientific, Waltham, MA, USA) at 37 °C and 5% CO_2_. HIV-1 IIIB-infected H9 cell line [61,62,63] was obtained from the NIH AIDS Research and Reference Reagent Program (NIH-ARRRP). The HIV-1 IIIB virus was harvested from these H9 cells. pCH040.c/2625, a full-length transmitted/founder (T/F) HIV-1 subtype B infectious molecular clone [64] and HEK 293T cells were obtained from NIH-ARRRP. The HIV-1 CH040 virus was produced by transfecting HEK 293T cells with the pCH040.c/2625 plasmid using polyethylenimine (PEI) as described previously [65]. Filipin, mouse monoclonal anti-actin antibody, DMSO, and MPA were purchased from Sigma (St. Louis, MO, USA). LysoSensor Green DND-189 was purchased from Thermo Fisher Scientific. LC3B antibody was purchased from Cell Signaling Technology (Danvers, MA, USA). Pepstatin A, leupeptin and E-64 were purchased from AG Scientific (San Diego, CA, USA). 

### 4.2. Source of Tissues as Well as Cervical Epithelial Cell Isolation and Culture

Female genital tract tissues used in this study were provided by the Cooperative Human Tissue Network (CHTN)-Western Division at Vanderbilt University Medical Center and by the Meharry Medical College Translational Pathology/Tissue Acquisition Shared Resource. Identifying details of the samples were removed. The sample collection protocol was approved by the Meharry Medical College IRB. The protocol for isolating primary cervical epithelial cells was adapted from the method established by Fichorova et al. [66]. Briefly, the specimen tissues were first washed three times with Hank’s balanced salt solution containing 10% penicillin-streptomycin (Thermo Fisher Scientific) and then cut into 0.5 × 0.5 cm blocks with a sterile scalpel. The tissue blocks were then placed with the cervical surface down onto a six-well plate with 200 µL of EpiGRO^TM^ Human Epidermal Keratinocyte Complete Culture Medium (Millipore, Burlington, MA, USA) and cultured at 37 °C with 5% CO_2_ for 2 h. After the 2 h incubation, fresh EpiGRO^TM^ medium (2 mL) was added for long-term culture. The medium was replaced every three days. The adherent cells were usually visible in 7–10 days. Under normal circumstances, the cells would become confluent in 14 days. Cells were passaged when the confluency reached 80–90%.

### 4.3. MPA, Lysosomal Inhibitors Cocktail Treatment and Viral Inoculation

Physiologically relevant concentration of MPA (1 nM) [39,40] was used to pretreat VK2 cells or primary cervical epithelial cells for 1 h. Control cells were treated with DMSO alone. The cells, the VK2 cells were exposed to either 60 ng p24 protein content of HIV-1 IIIB or 200 ng p24 protein content of the CH040 virus for the indicated time periods. The primary cervical epithelial cells were exposed to 200 ng p24 protein content of the CH040 virus for the indicated time periods. After viral exposure, the virus-containing medium was removed. Trypsin (0.03%) was used to treat the cells at room temperature for 1 min to remove viral particles attached to the cell surface. After three washes with PBS, the cells were cultured in fresh medium containing 1 nM MPA for the indicated time periods. The culture medium and cells were collected at the indicated time points and subjected to qRT-PCR and Western blot analysis, respectively.

Lysosomal inhibitors cocktail containing pepstain A, leupeptin, and E-64 was used to pretreat VK2 cells overnight at the final concentration of 29 µM pepstatin A, 52 mM leupeptin and 69 mM E-64. One-hour 1 nM MPA treatment was added into the overnight treated samples for the combination treatment of MPA and lysosomal inhibitors cocktail. After the pretreatment, the cells were exposed to CH040 virus as described above. 

### 4.4. SDS-PAGE and Western Blot

The harvested cells were lysed using RIPA buffer (10 mM Tris-Cl [pH 8.0] containing 1 mM EDTA, 0.5 mM EGTA, 1% Triton X-100, 0.1% sodium deoxycholate, 0.1% SDS, 140 mM NaCl, and fresh 1 mM PMSF). The cell lysates were subjected to a 21-kg spin at 4 °C for 1 h. The supernatant was mixed with 4×SDS-PAGE sample buffer (Bio-Rad, Hercules, CA, USA) and subjected to SDS-PAGE. The resultant protein bands were transferred to Amersham Protran Premium 0.2 µm NC (Sigma) using the Bio-Rad Trans-Blot Turbo Transfer System. Anti-HIV-1 p24 monoclonal antibody (NIH-ARRRP, 183-H12-5C) [67], Polyclonal Anti-Human Immunodeficiency Virus Immune Globulin (NIH-ARRRP, ARP-395), and anti-actin (Sigma) were used for staining IIIB p24, CH040 p24 and action respectively in Western blotting as previously described [68]. Bio-Rad ChemiDoc MP was used to visualize the protein bands. 

### 4.5. LysoSensor^TM^ Staining

Primary cervical epithelial cells were treated as described earlier. After the 1 h MPA treatment, the cells were washed twice with PBS. LysoSensor Green DND-189 (0.5 µM) (Thermo Fisher Scientific) was then used to stain the cells at 37 °C for 15 min following manufacturer’s instructions. After staining, the cells were washed twice with PBS and then analyzed using BD FACSCalibur (BD Biosciences, San Jose, CA, USA). The LysoSensor Green DND-189 staining of the primary cervical epithelial cell samples was duplicated, and the mean of the MFI was used for the data processing. 

### 4.6. Transfection and Flow Cytometry Analysis

The LC30-GFP fusion protein expression plasmid pEX-GFP-hLC3WT was transfected into the primary cervical epithelial cells using Lipofectamine 2000 (Themo Fisher Scientific) following manufacturer’s instructions. pEX-GFP-hLC3WT [43] was a gift from Dr. Isei Tanida (Addgene plasmid # 24987). Twenty-four hours after transfection, the cells were treated with 1 nM MPA dissolved in DMSO for 1 h. Control cells were treated with DMSO alone. After the treatment, the cells were detached using trypsin, washed twice with PBS, and subjected to flow cytometry analysis using BD FACSCalibur^TM^.

### 4.7. qRT-PCR

qRT-PCR was used to measure relative viral release. Total RNA from the viral samples was isolated using the Aurum total RNA Mini Kit (Bio-Rad). cDNA was produced using the High Capacity cDNA Reverse Transcription Kit (Themo Fisher Scientific). qPCR was performed on a Bio-Rad CFX96 Touch Real-Time PCR Detection System using the following program: 95 °C (3 min); 40 cycles of 95 °C (15 s); 60 °C using SYBR Green Supermix (Bio-Rad) (1 min). The primers used were LTR S4 (5’-AAGCCTCAATAAAGCTTGCCTTGA) and LTR AS3 (5’-GTTCGGGCGCCACTGCTAG), as described previously [69]. Each pair of DMSO and MPA samples was analyzed in the same qPCR run. Relative viral release was calculated using Bio-Rad CFX Maestro based on the 2^−ΔΔCT^ method. Paired *t*-test was used to calculate the p-value. 

### 4.8. Statistical Analysis

Paired *t*-test was performed using GraphPad Prism. A *p*-value of ≤0.05 was considered statistically significant (*). A *p*-value of ≤0.01 was considered statistically very significant (**).

## 5. Conclusions

In this study, we show that MPA treatment of human primary cervical/vaginal epithelial cells led to a reduction in lysosomal activity, consequently resulting in increased intracellular HIV-1 accumulation and virus release with implications of higher viral transmission rates. These data suggest a novel mechanism by which MPA influences HIV susceptibility.

## Data Availability

The data presented in this study are available in the article and its Supplementary Material.

## Supplementary Materials

The following are available online at https://www.mdpi.com/article/10.3390/pathogens10091192/s1.
