# Supplementary material for: Medroxyprogesterone Acetate (MPA) Enhances HIV-1 Accumulation and Release in Primary Cervical Epithelial Cells by Inhibiting Lysosomal Activity
Source: Pathogens. 2021 Sep 14;10(9):1192. doi: 10.3390/pathogens10091192 (PMC8465616; doi:10.3390/pathogens10091192)
Supplement: Supplementary file 1 [file pathogens-10-01192-s001.zip › pathogens-1326294-supplementary.pdf]

Fig S1

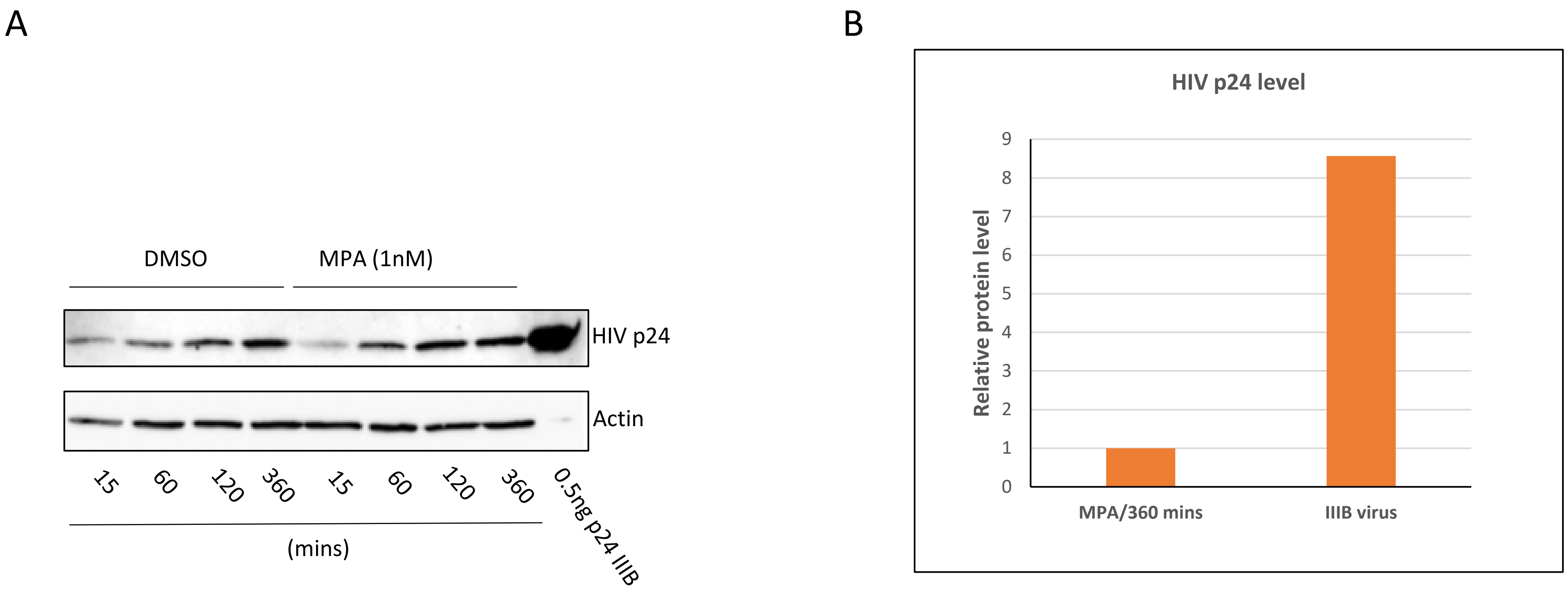

Fig S1. The efficiency of HIV-1 IIIB uptake by VK2 cells. (A) 0.5ng p24 content of HIV-1 IIIB virus was subjected to western blot analysis along with one group of sample from Fig 1A. (B) The relative protein level between MPA treated 360 mins sample and the 0.5ng p24 content of HIV-1 IIIB virus was analyzed by densitometry analysis. 60 ng p24 content of IIIB was exposed to the VK2 cells. The estimated efficiency of HIV-1 IIIB uptake by the VK2 cell (treated by MPA for 360 mins) equals: (0.5/8.5)/60=~0.1%.

Original western blot imagines

Fig 1A

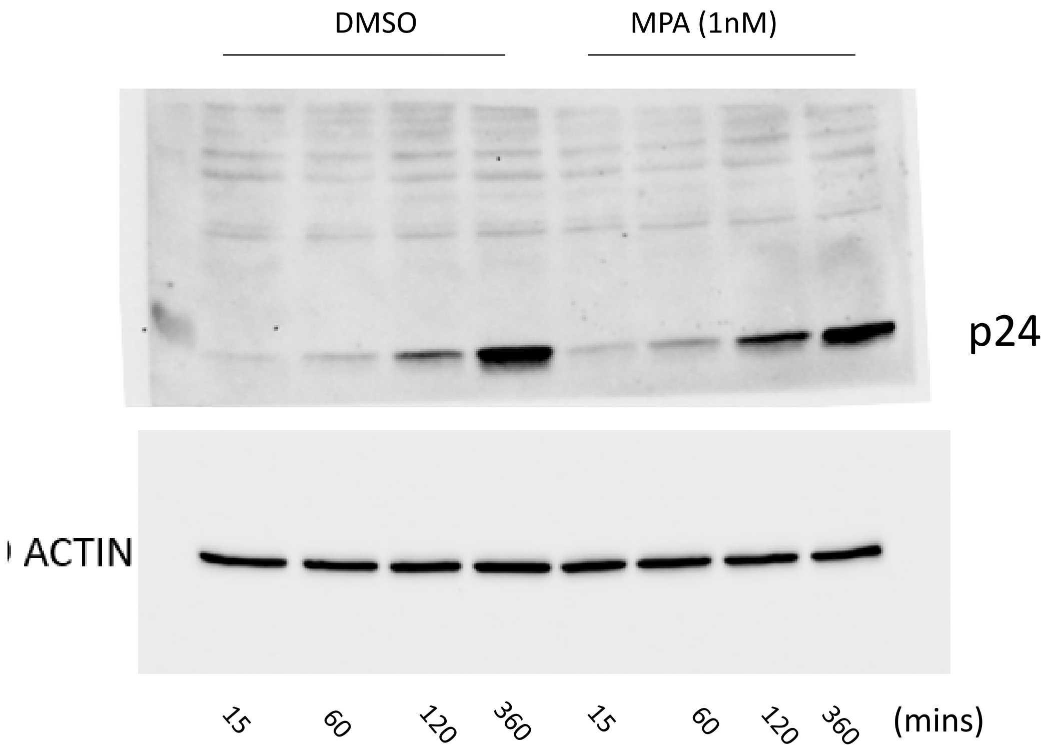

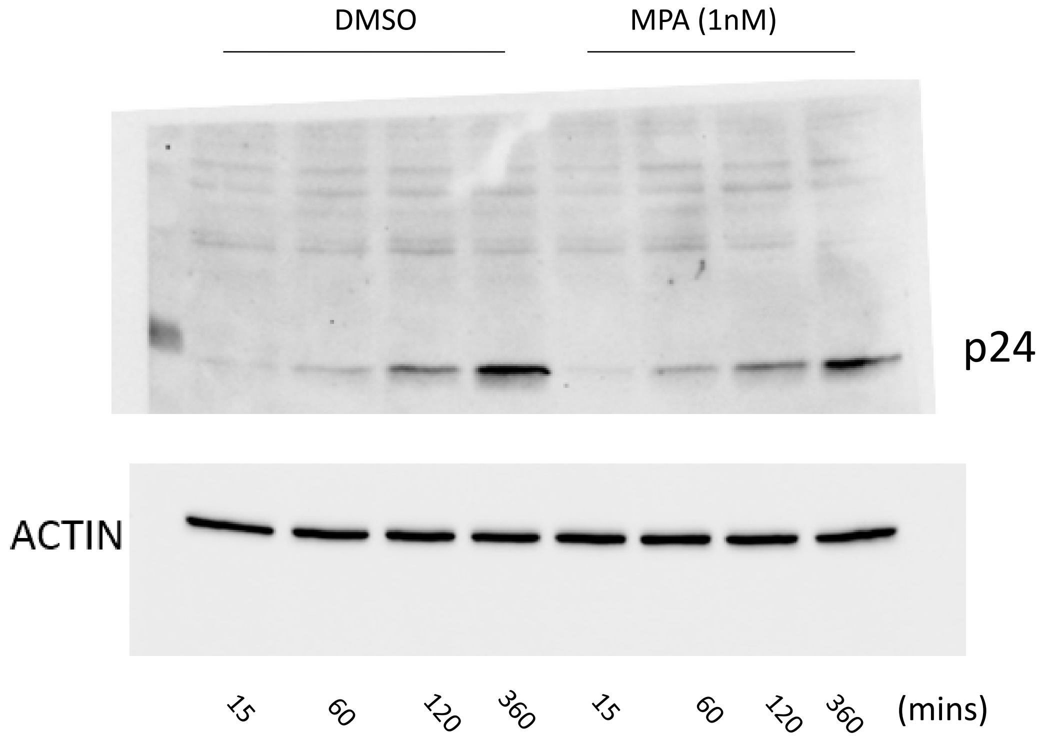

Fig 1A

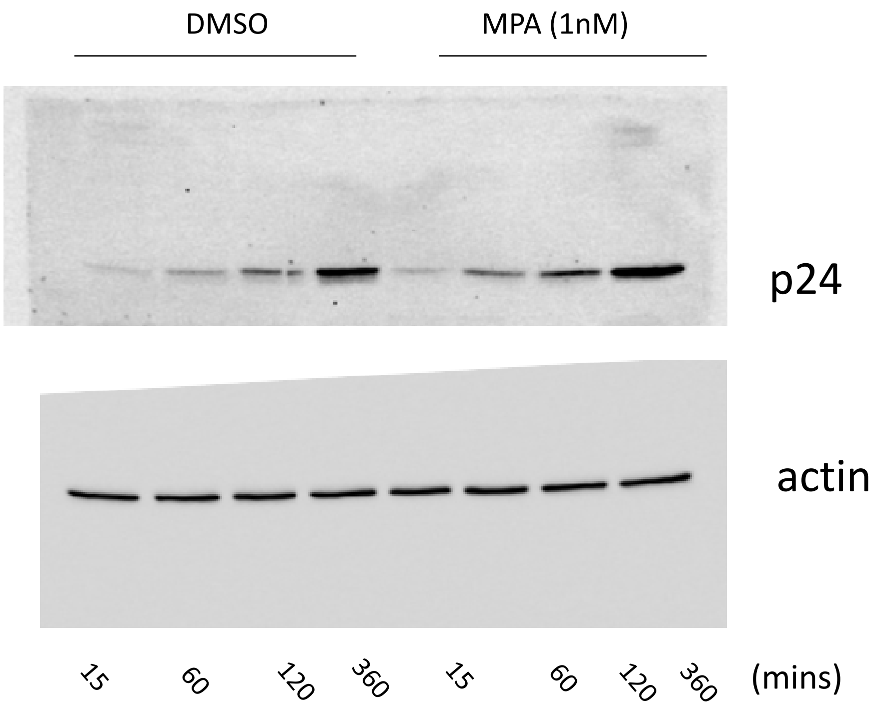

Fig 1B

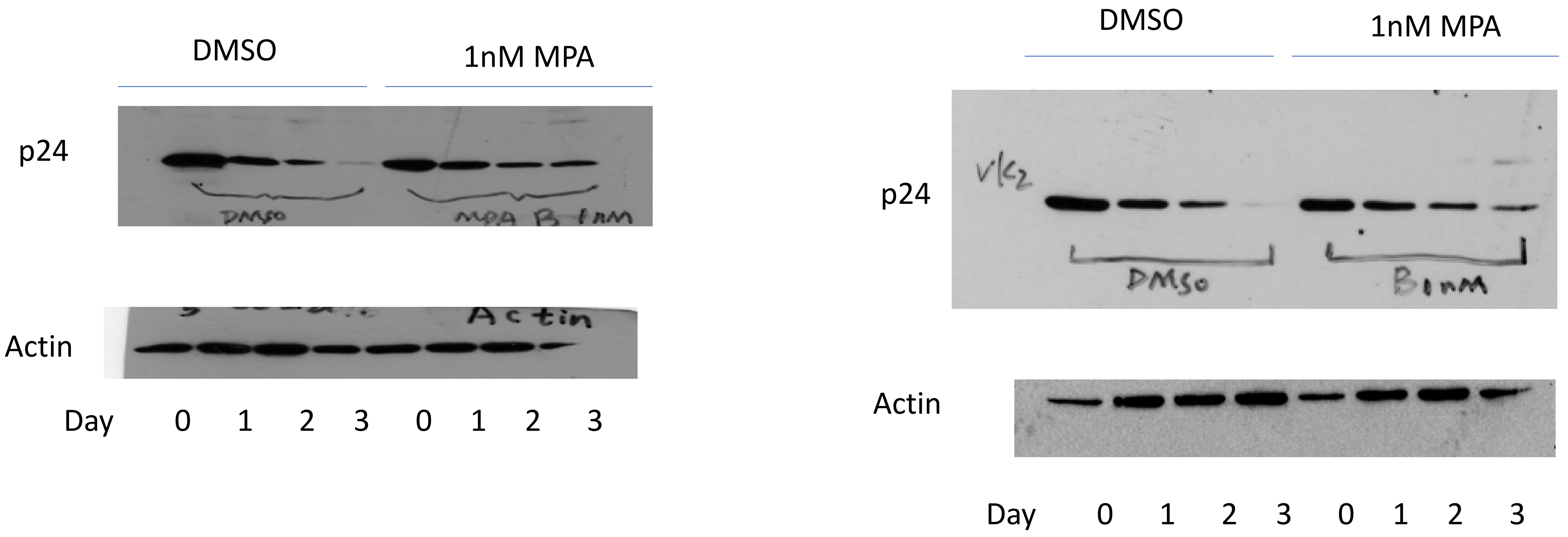
DMSO
1nM MPA
p24
Actin
Day
0 1 2 3 0 1 2 3
DMSO
1nM MPA
p24
Actin
Day
0 1 2 3 0 1 2 3

Fig 1B

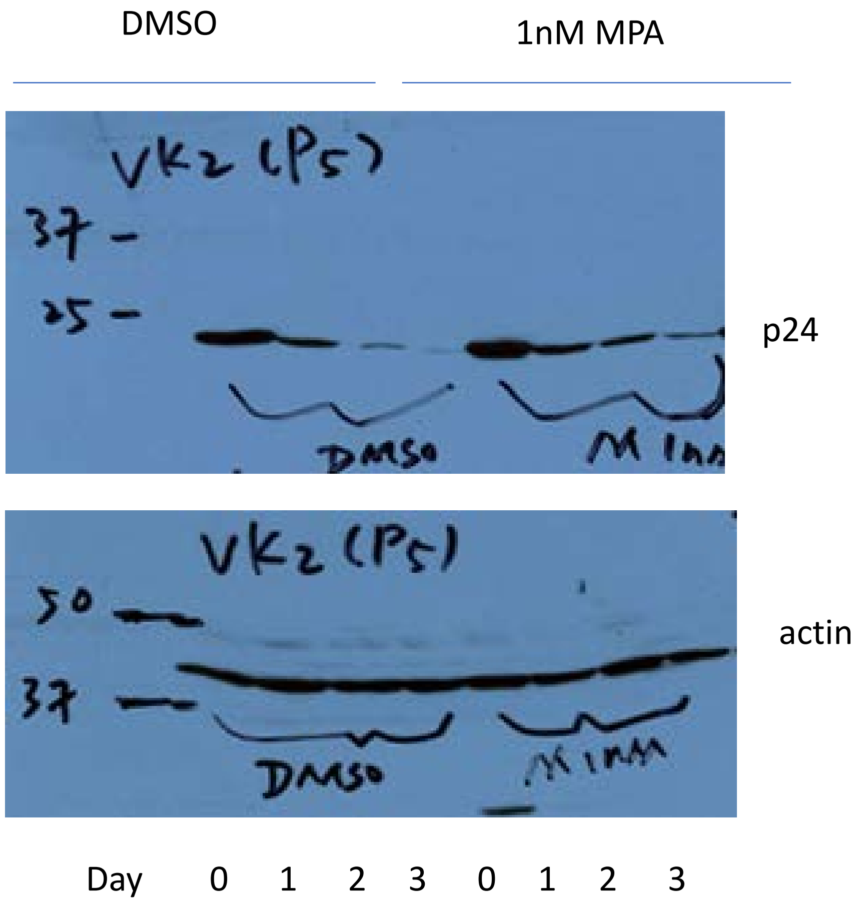
DMSO
1nM MPA
VK2 (P5)
37
25
DMSO
p24
VK2 (P5)
50
37
DMSO
actin
Day 0 1 2 3 0 1 2 3

Fig 2A-1

DMSO 1nM MPA

Donor 811

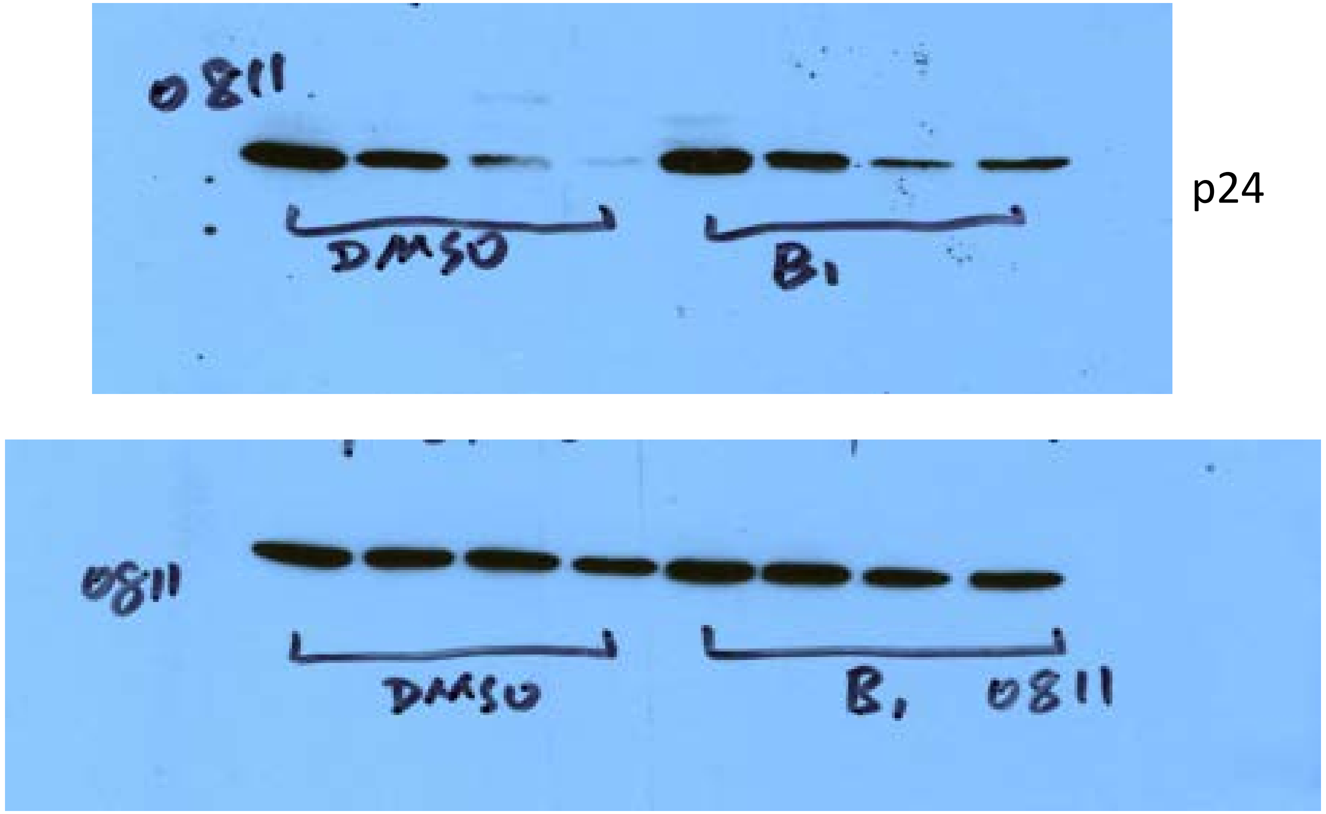

p24

Actin

Day 0 1 2 3 0 1 2 3

Fig 2A-2

DMSO 1nM MPA

Donor 1003

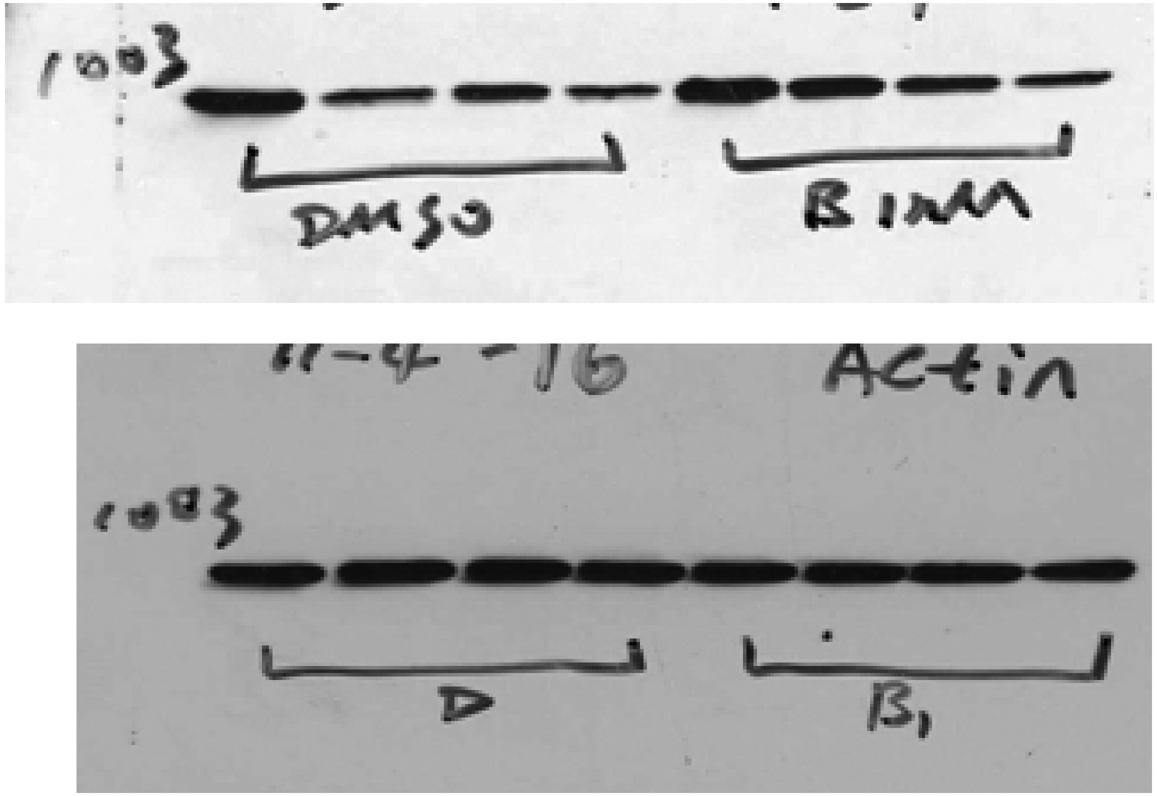

p24

Day 0 1 2 3 0 1 2 3

Fig 2B

DMSO 1nM MPA

Donor 817

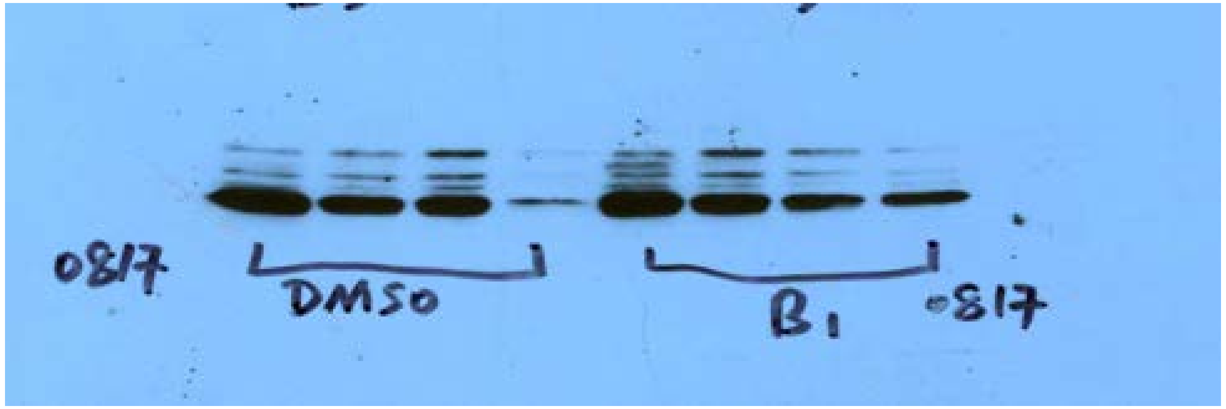

p24

Day 0 1 2 3 0 1 2 3

Fig 2B

Donor 0513

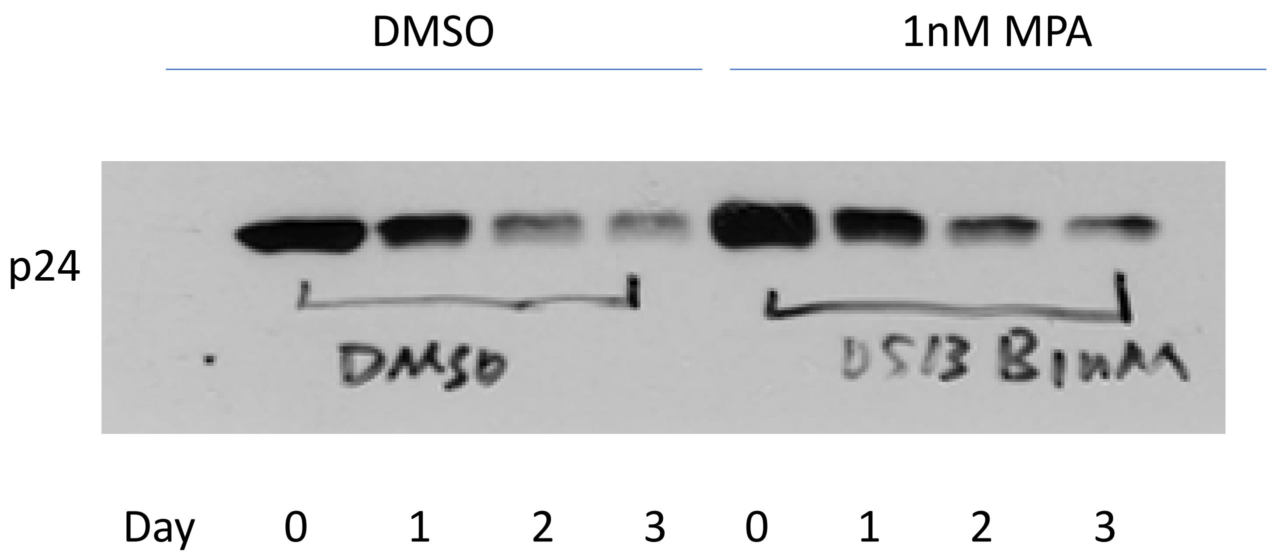

Fig 2B

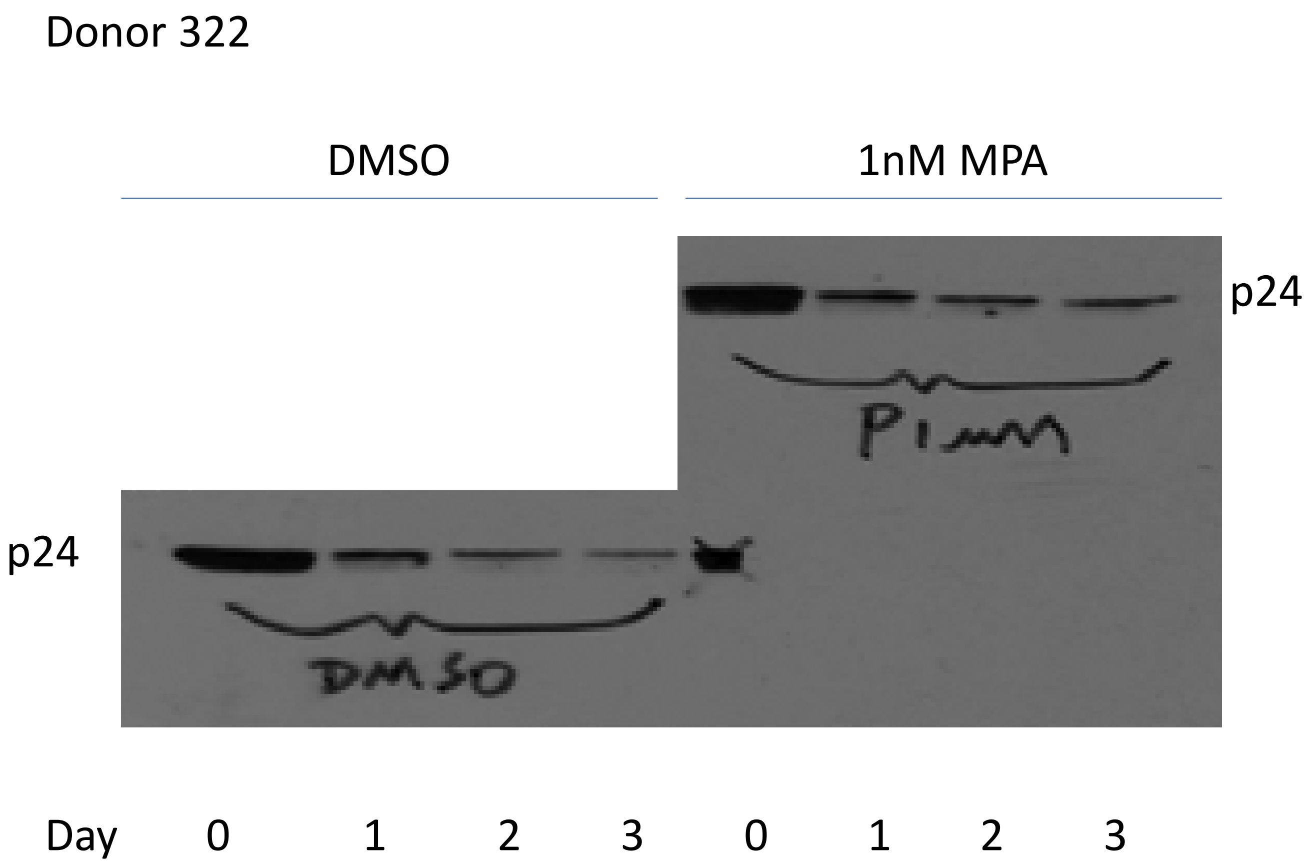

Fig 2B

DMSO 1nM MPA

Donor 714

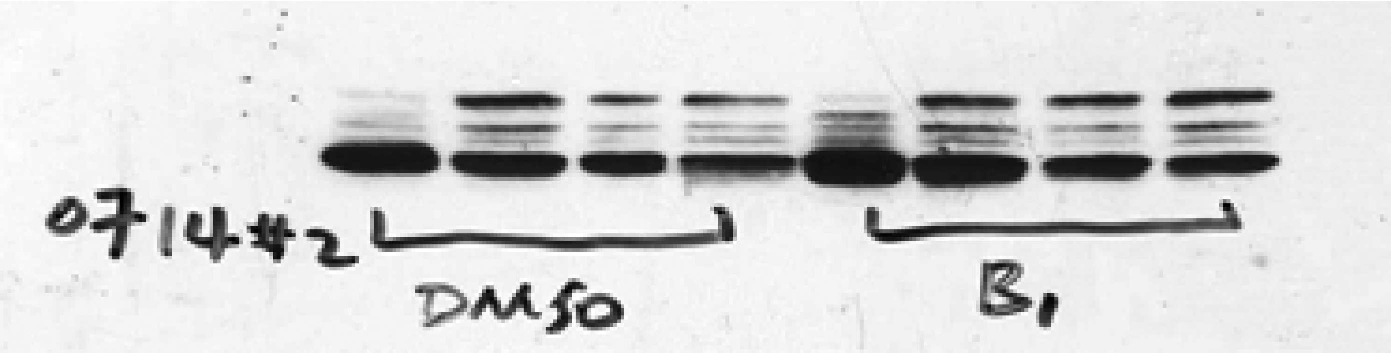

p24

Day 0 1 2 3 0 1 2 3

Fig 2B

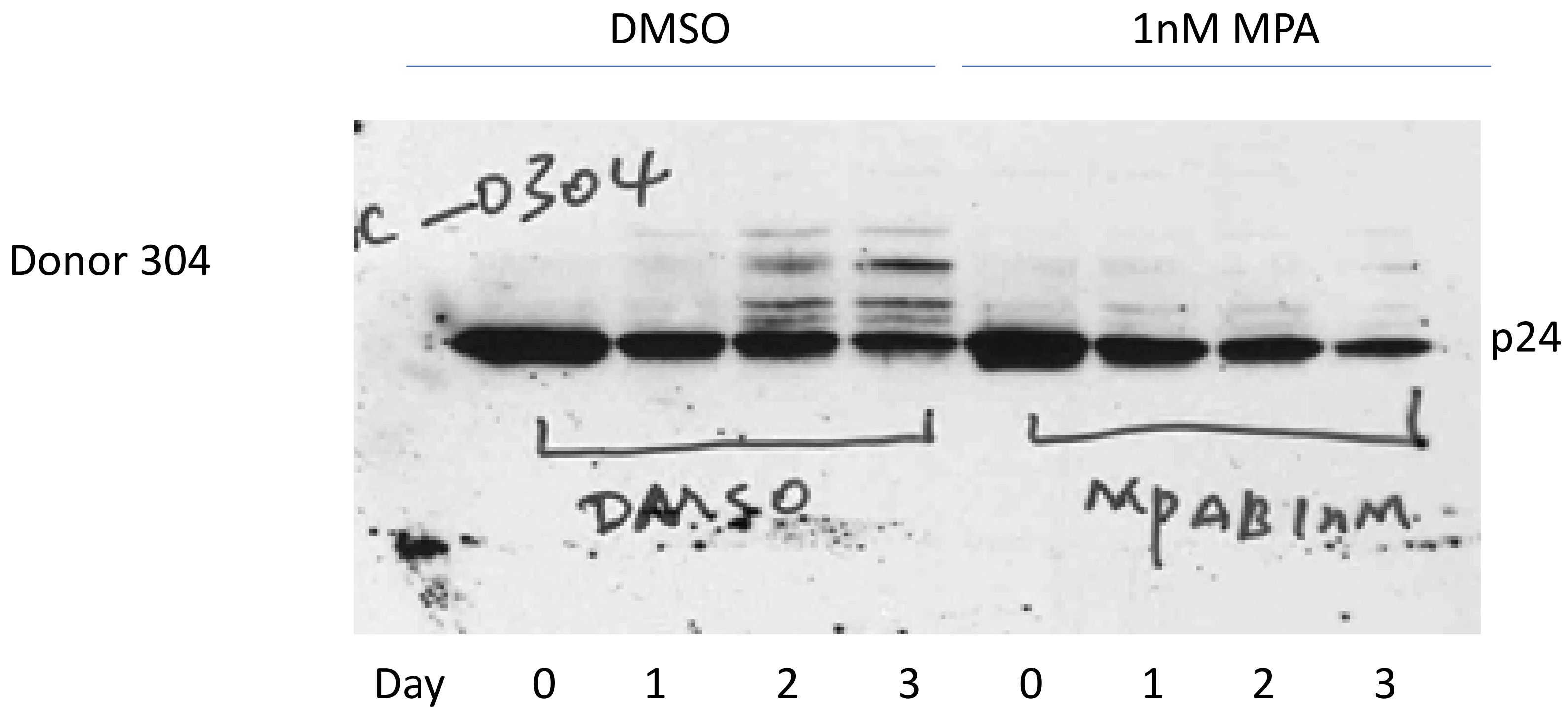

Fig 2B

DMSO 1nM MPA

Donor 601

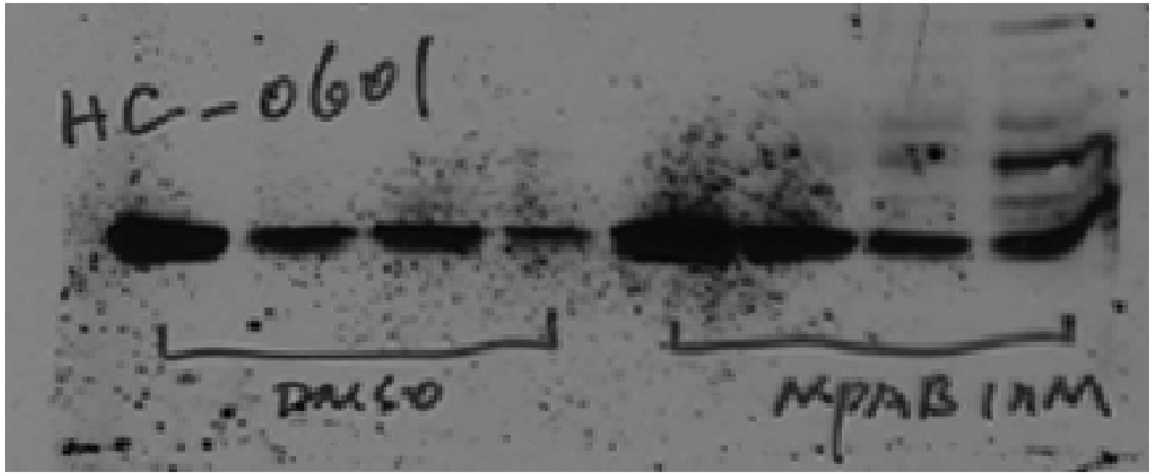

p24

Day 0 1 2 3 0 1 2 3

Fig 2B

	DMSO	1nM MPA	

Donor 1005

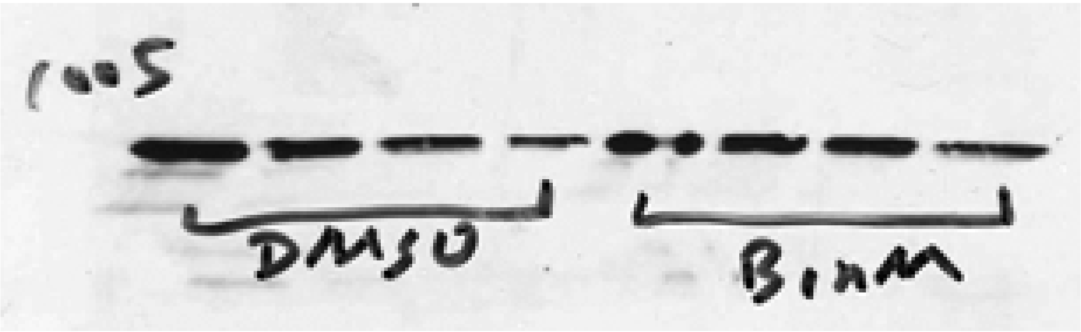

p24

Day	0	1	2	3	0	1	2	3

Fig 2B

DMSO 1nM MPA

Donor 0923

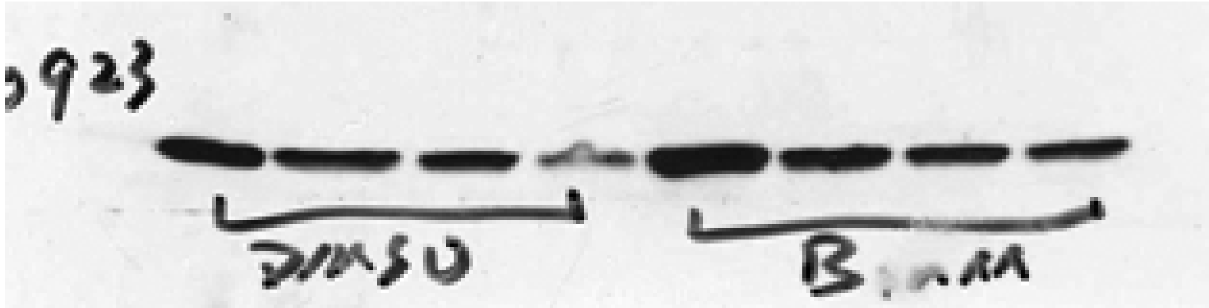

p24

Day 0 1 2 3 0 1 2 3

Fig 2B

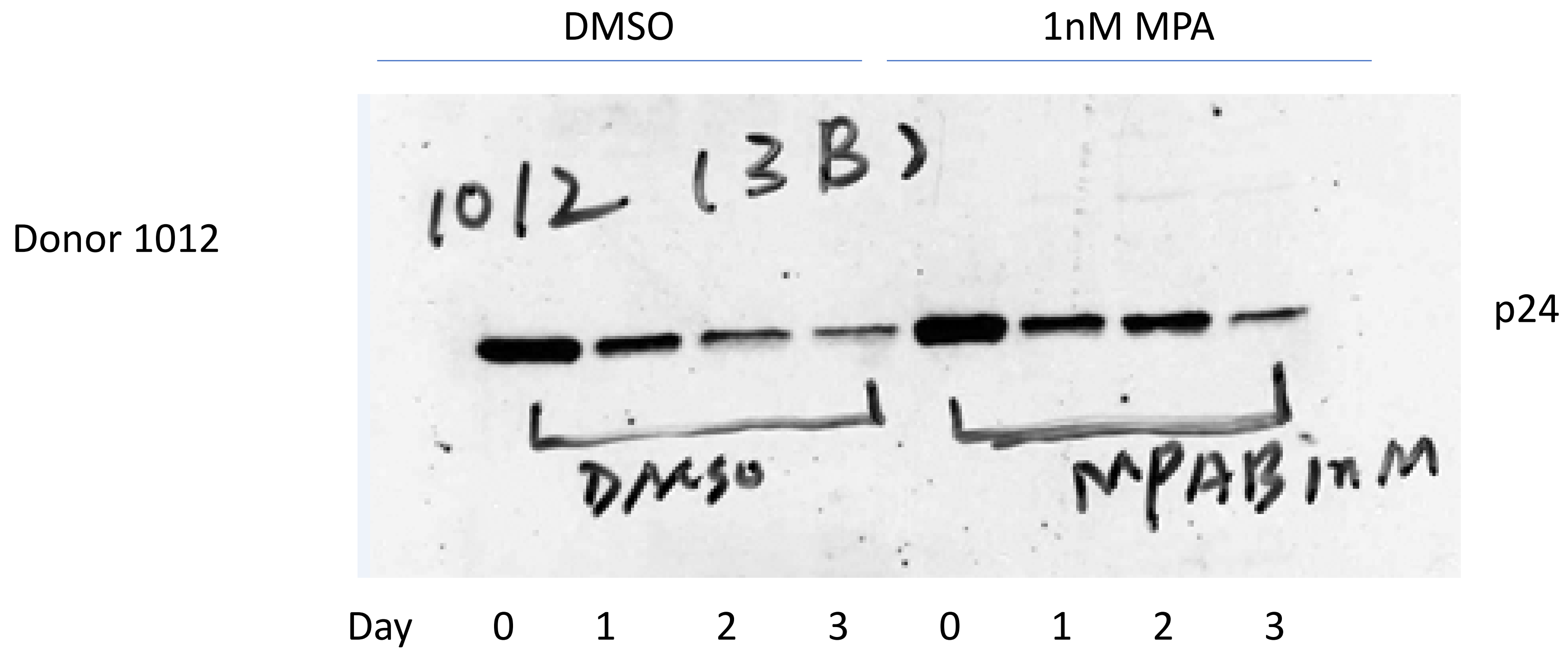
DMSO
1nM MPA
Donor 1012
1012 (3B)
DMSO
MPAB 1nM
p24
Day 0 1 2 3 0 1 2 3

Fig 2B

DMSO | 1nM MPA

Donor HV

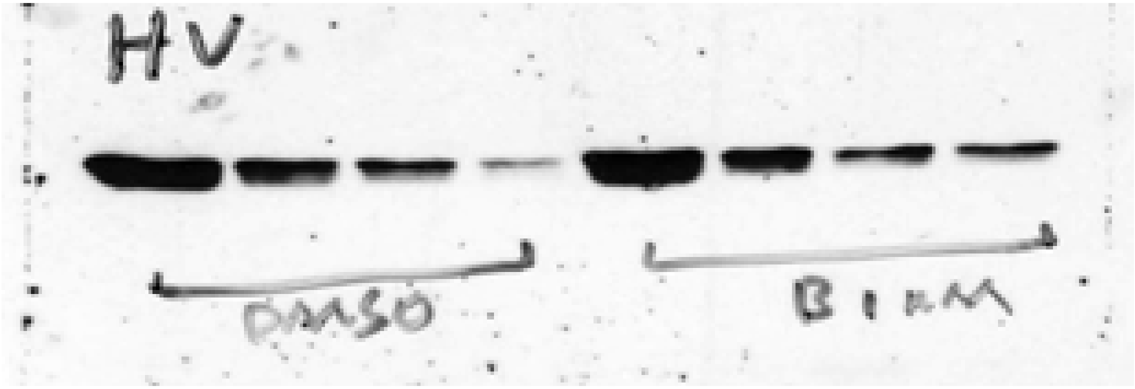

p24

Day 0 1 2 3 0 1 2 3

Fig 5A

1st time

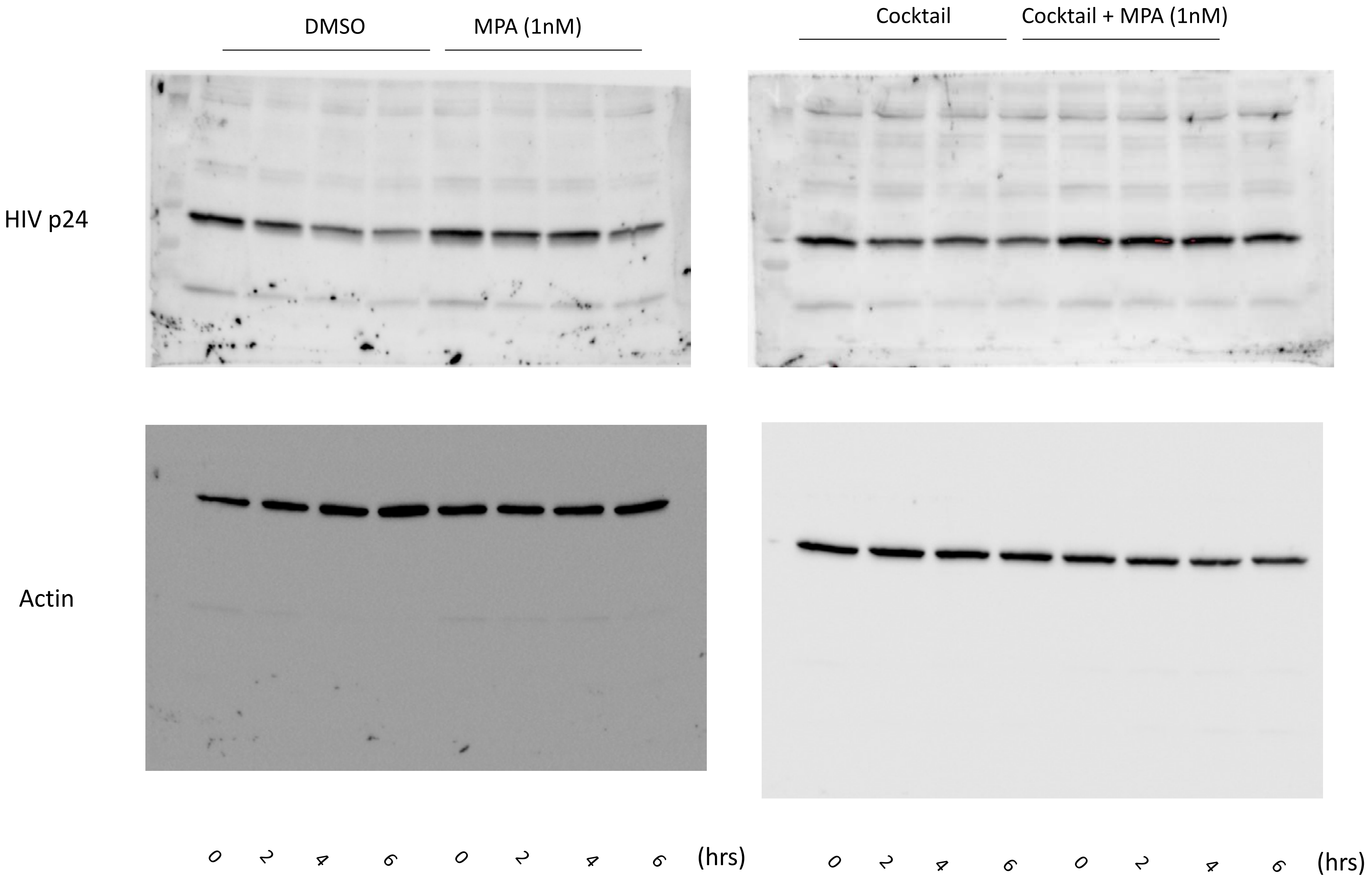

Fig 5A

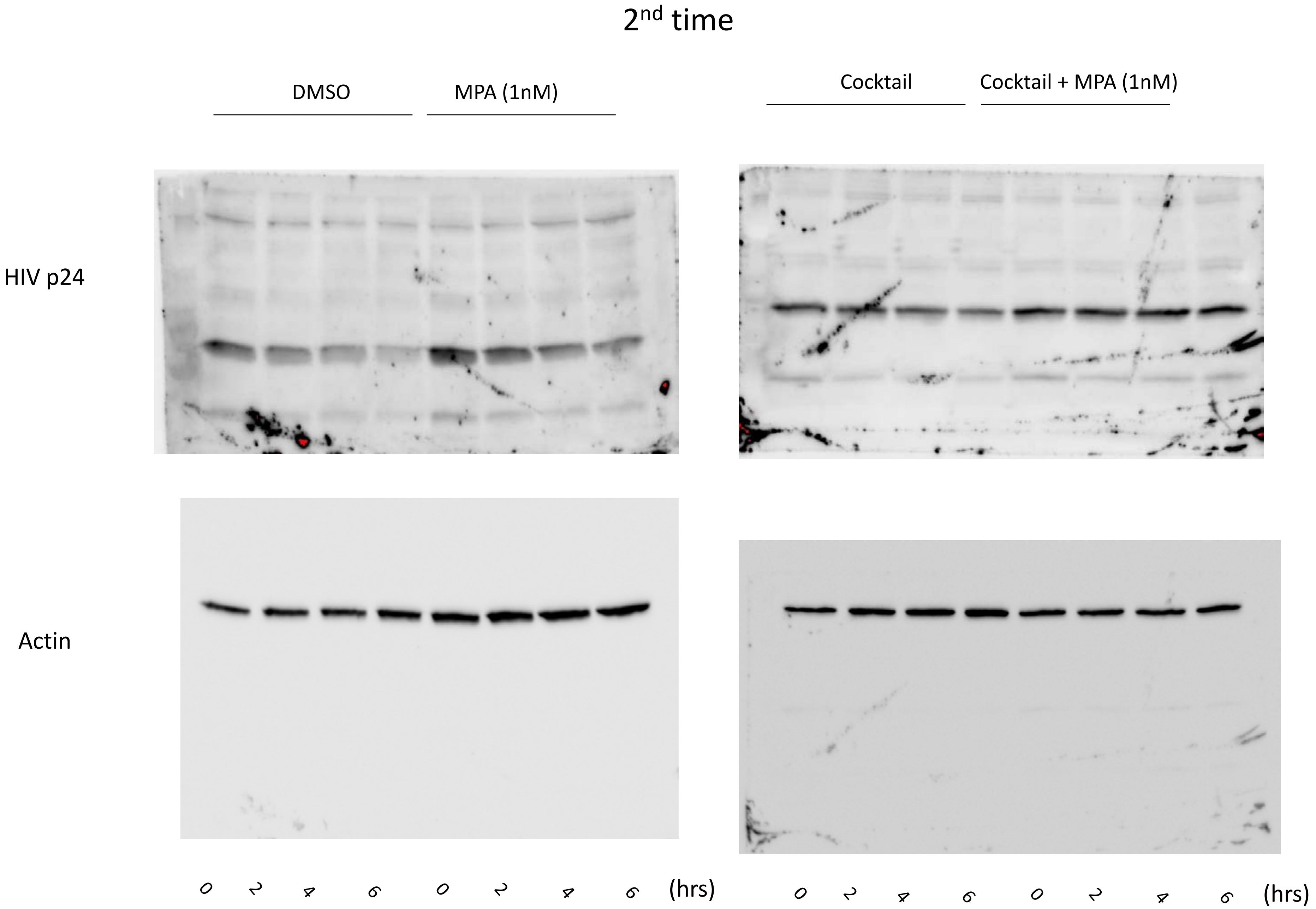
2nd time
DMSO
MPA (1nM)
Cocktail
Cocktail + MPA (1nM)
HIV p24
Actin
0
2
4
6
0
2
4
6
(hrs)
0
2
4
6
0
2
4
6
(hrs)

Fig 5A left panel

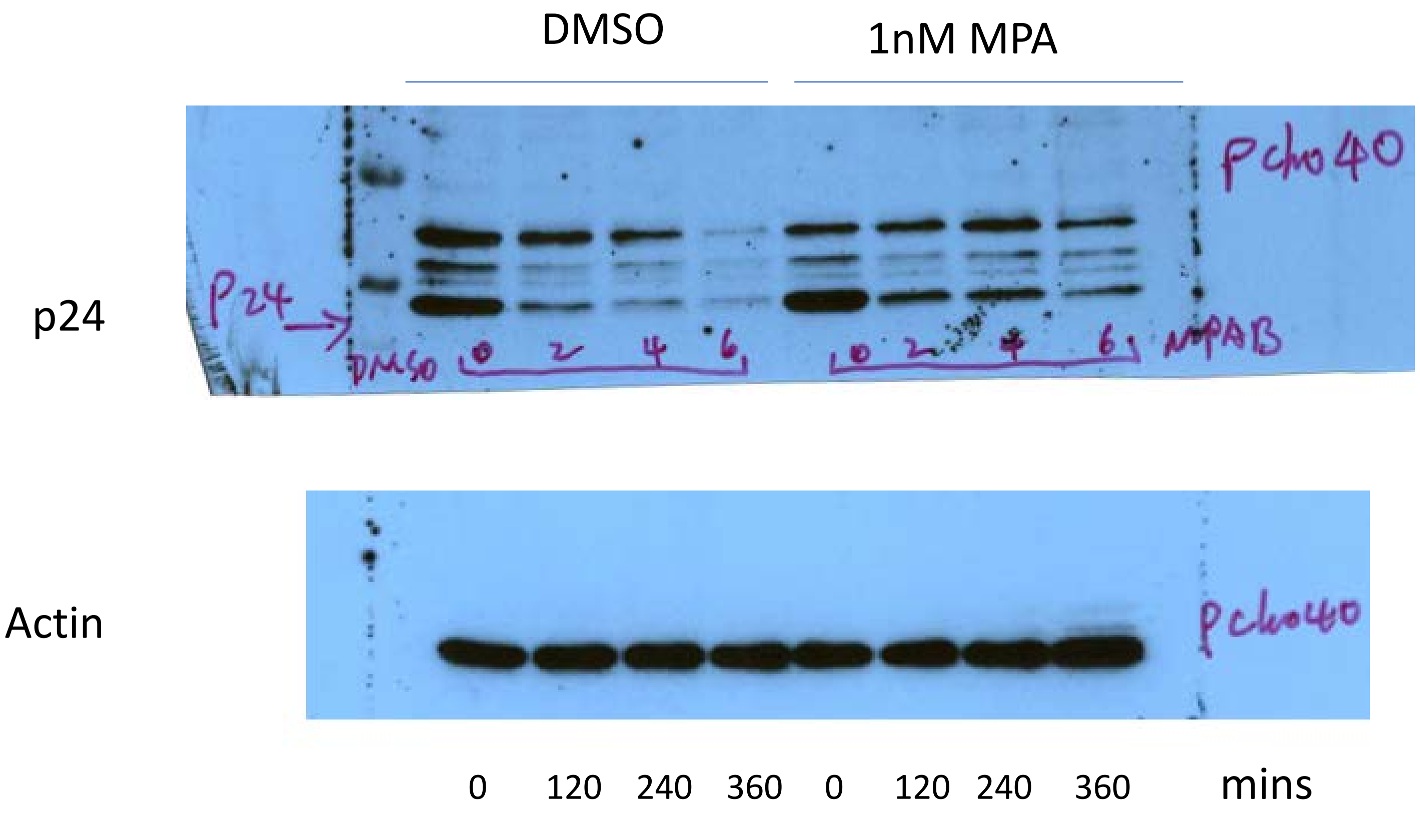

Fig S1

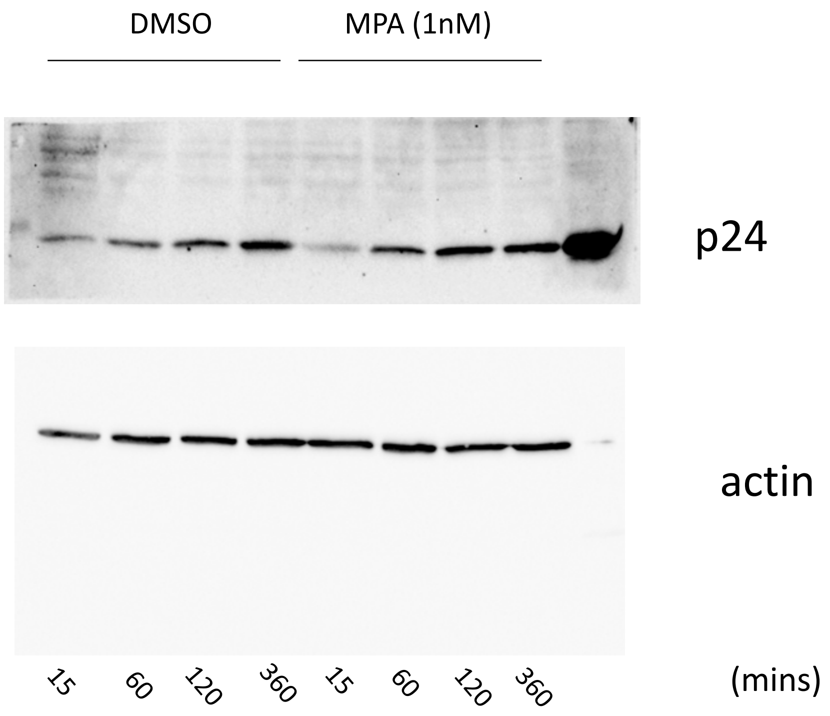

Original data for Figure 3

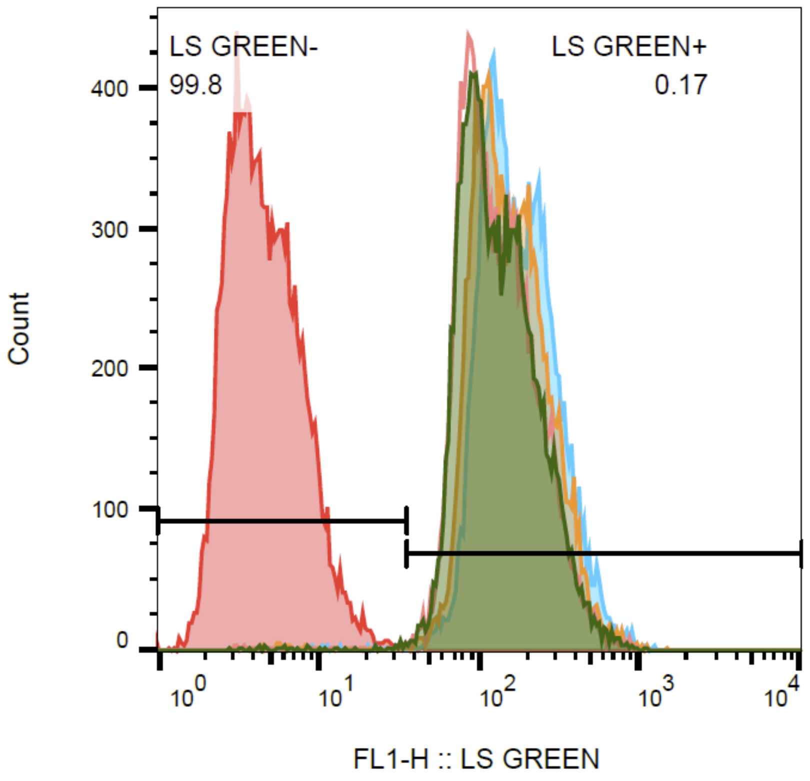

	Sample Name	Subset Name	Count	Geometric Mean : FL1-H
	0113 MPAB -.005	LIVE	14327	132
	0113 MPAB +.004	LIVE	14504	131
	0113 DMSO.003	LIVE	14322	154
	0113 UT.002	LIVE	14407	175
	0113 US.001	LIVE	14429	4.71

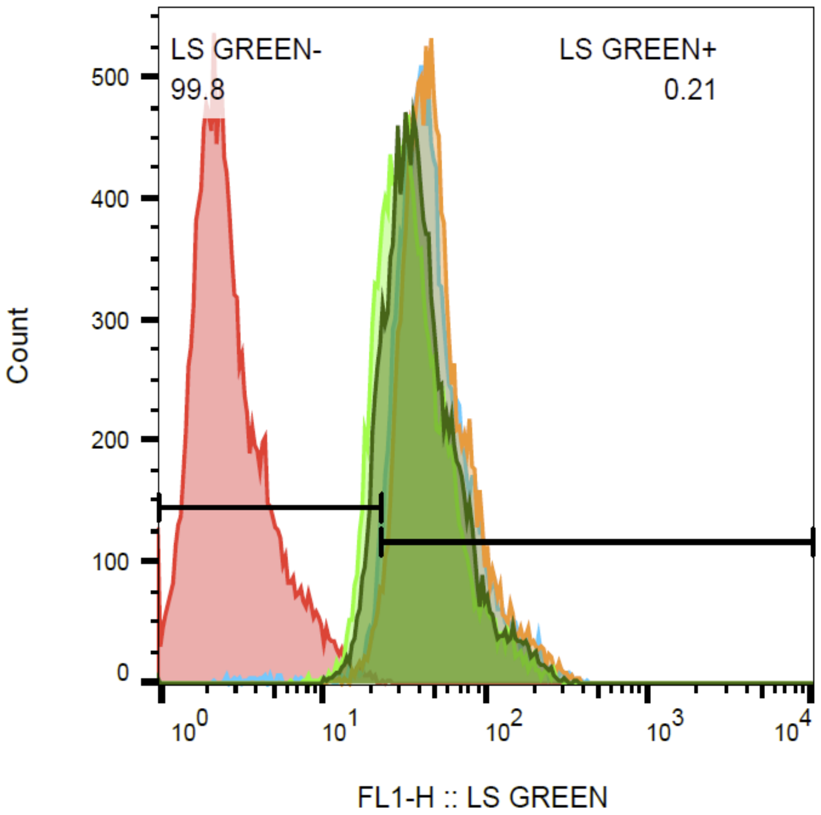

	Sample Name	Subset Name	Count	Geometric Mean : FL1-H
	HT 0127 MPAB B.005	LIVE	14809	40.9
	HT 0127 MPAB A.004	LIVE	14816	36.8
	HT 0127 DMSO.003	LIVE	14823	51.0
	HT 0127 UT.002	LIVE	14840	47.0
	HT 0127.001	LIVE	14747	2.82

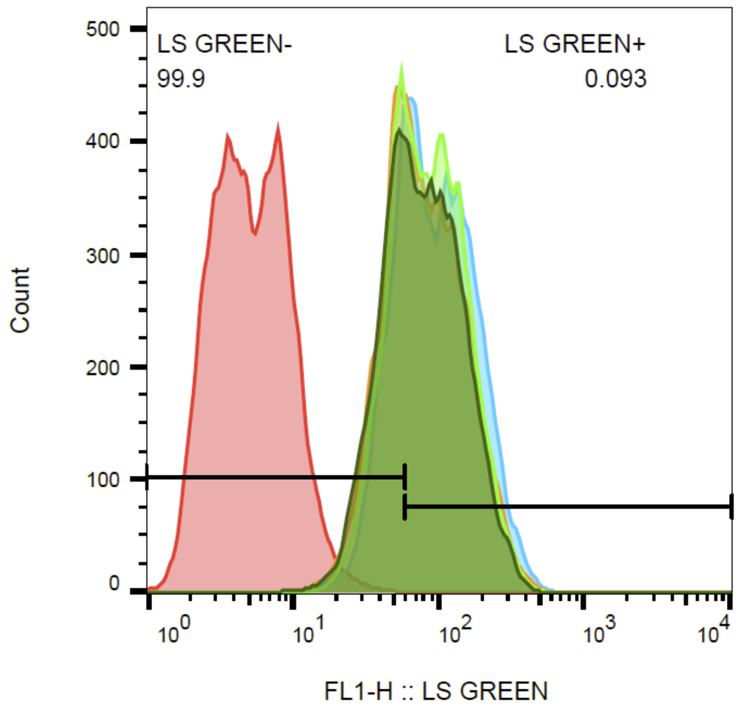

	Sample Name	Subset Name	Count	Geometric Mean : FL1-H
	HT0202 mpab b.005	LIVE	19001	77.2
	HT0202 mpab a.004	LIVE	19693	84.4
	HT0202 dmso.003	LIVE	19568	79.9
	HT0202 ut.002	LIVE	19704	93.4
	HT0202.001	LIVE	19343	5.27

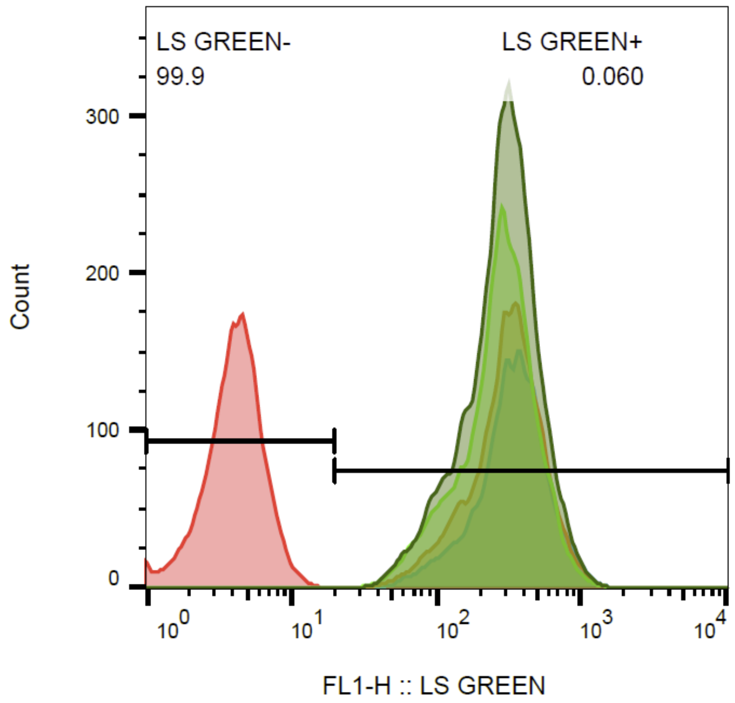

	Sample Name	Subset Name	Count	Geometric Mean : FL1-H
	HT0202 #2 MPAB b.005	LIVE	10804	274
	HT0202 #2 MPAB a.004	LIVE	8077	261
	HT0202 #2 DMSO.003	LIVE	6247	298
	HT0202 #2 UT.002	LIVE	5156	324
	HT0202 #2 US.001	LIVE	5029	4.09

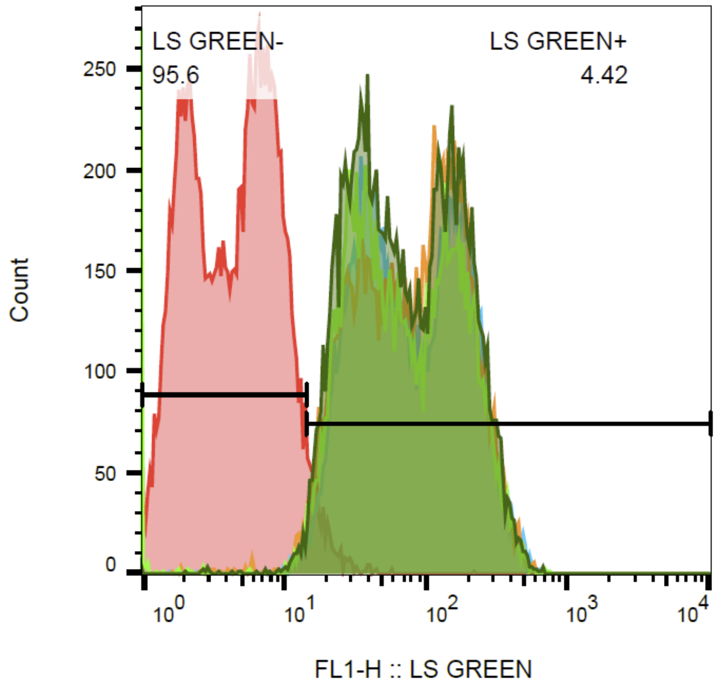

	Sample Name	Subset Name	Count	Geometric Mean : FL1-H
	HT 0203 MPAB a.004	LIVE	14529	72.2
	HT 0203 MPAB b.005	LIVE	12678	54.3
	HT 0203 DMSO.003	LIVE	12834	77.7
	HT 0203 UT.002	LIVE	12039	78.3
	HT 0203 US.001	LIVE	13739	4.40

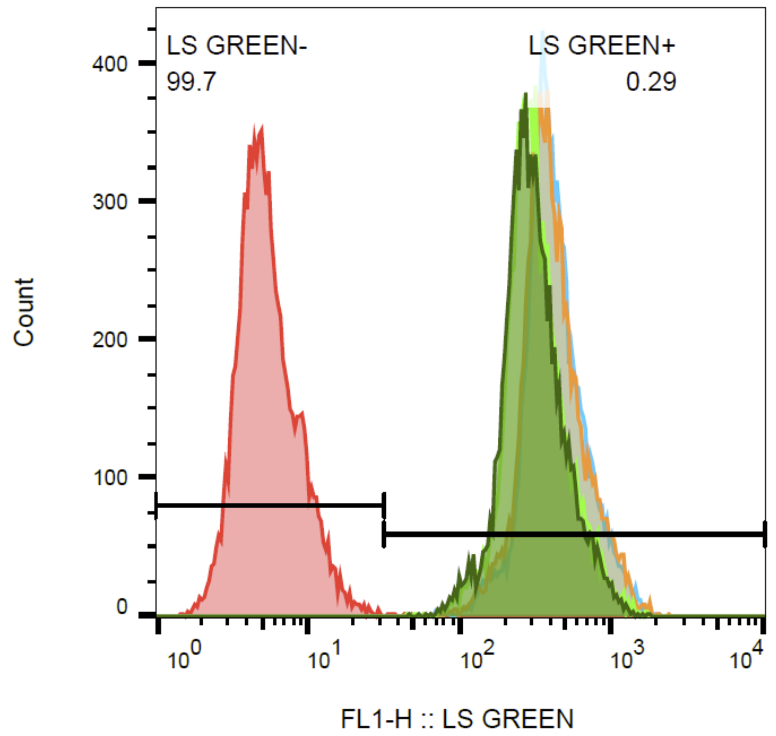

	Sample Name	Subset Name	Count	Geometric Mean : FL1-H
	HT0208#1 MPAB b.005	LIVE	9773	305
	HT0208#1 MPAB a.004	LIVE	9797	318
	HT0208#1 DMSO.003	LIVE	9756	407
	HT0208#1 UT.002	LIVE	9796	418
	HT0208#1 US.001	LIVE	9697	5.58

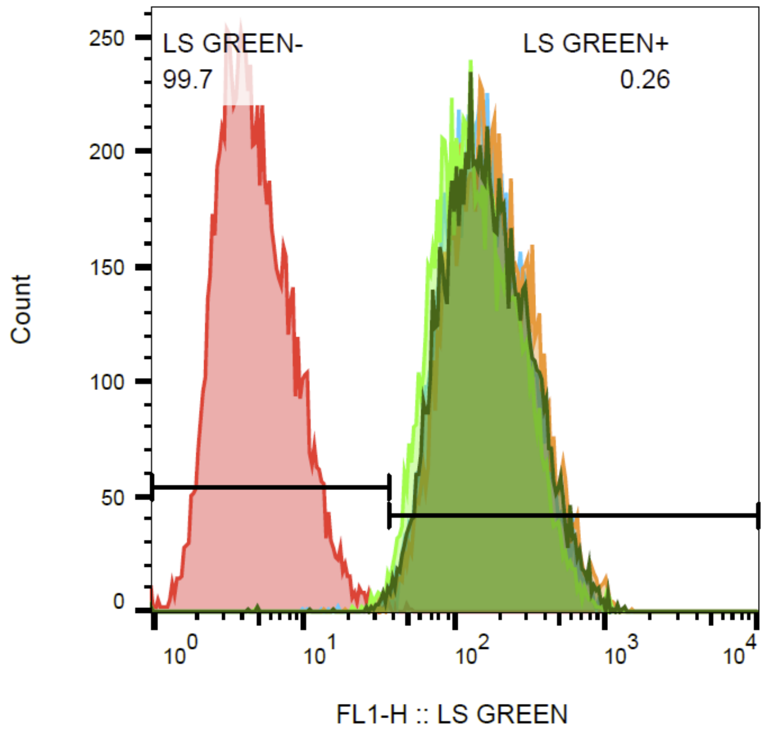

	Sample Name	Subset Name	Count	Geometric Mean : FL1-H
	HT0304 MPAB -.005	LIVE	9824	161
	HT0304 MPAB +.004	LIVE	9841	139
	HT0304 DMSO.003	LIVE	9881	175
	HT0304 UT.002	LIVE	9876	158
	HT0304 US.001	LIVE	9646	4.86

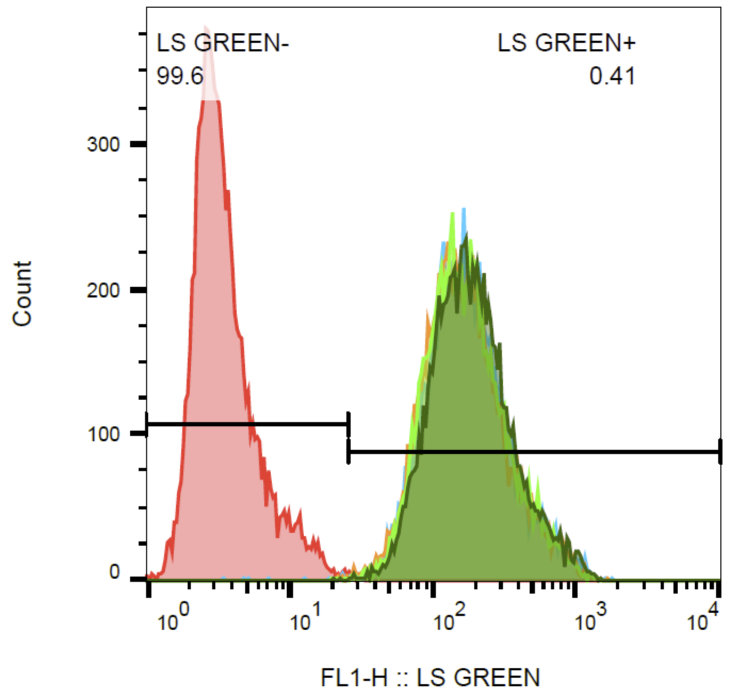

	Sample Name	Subset Name	Count	Geometric Mean : FL1-H
	HT0306 #1 MPA B b.005	LIVE	9808	186
	HT0306 #1 MPA B a.004	LIVE	9840	170
	HT0306 #1 DMSO.003	LIVE	9823	166
	HT0306 #1 UT.002	LIVE	9829	172
	HT0306 #1 US.001	LIVE	9811	3.64

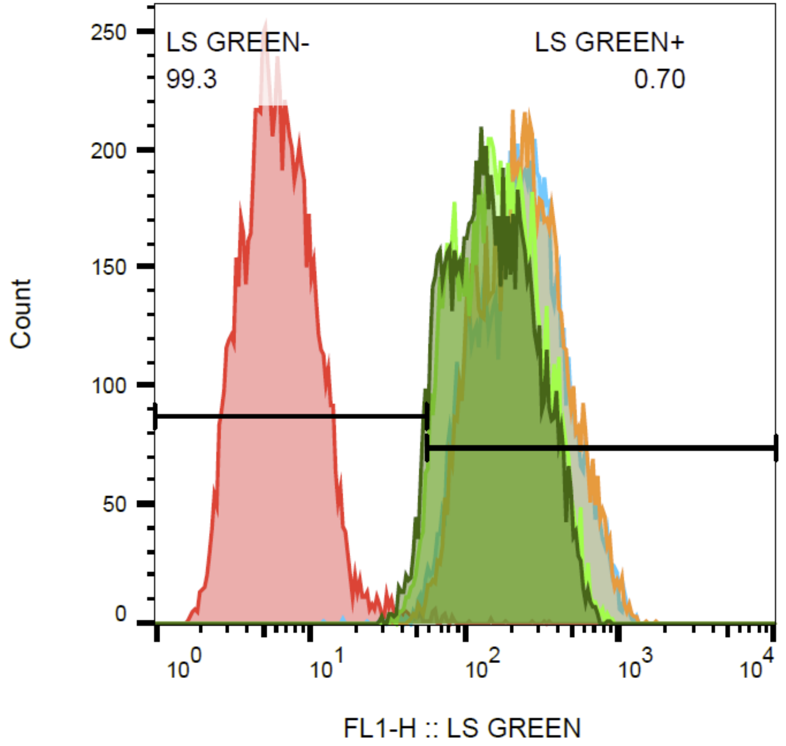

	Sample Name	Subset Name	Count	Geometric Mean : FL1-H
	HT0322 MPAB -.010	LIVE	9554	149
	HT0322 MPAB +.009	LIVE	9583	162
	HT0322 DMSO.008	LIVE	9492	233
	HT0322 UT.007	LIVE	9227	226
	HT0322 US.006	LIVE	9265	6.54

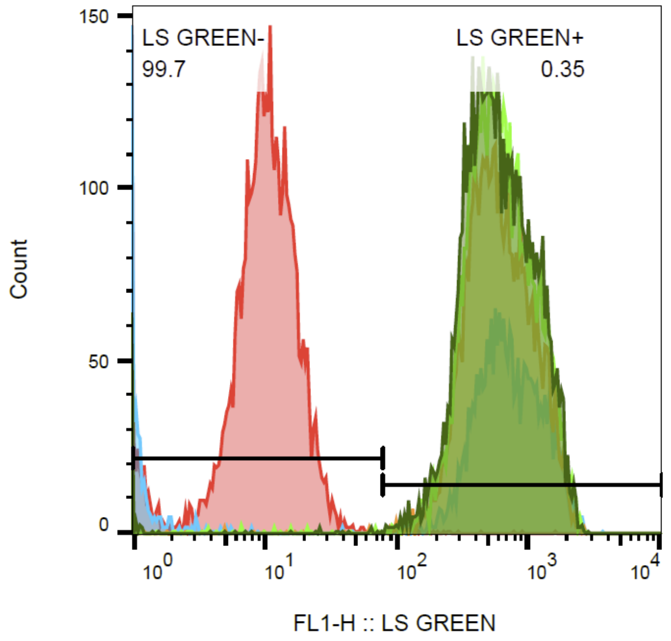

	Sample Name	Subset Name	Count	Geometric Mean : FL1-H
	0330 MPA-.005	LIVE	5833	541
	0330 MPA+.004	LIVE	5379	596
	0330 DMSO.003	LIVE	4450	588
	0330 UT.002	LIVE	4045	50.0
	0330 US.001	LIVE	5733	5.99

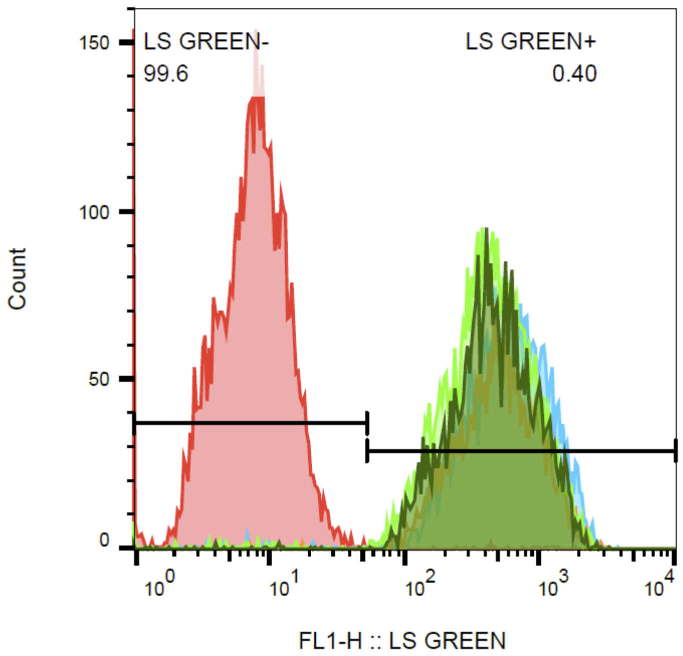

	Sample Name	Subset Name	Count	Geometric Mean : FL1-H
	0406 MPAB-.010	LIVE	3688	438
	0406 MPAB+.009	LIVE	4471	398
	0406 DMSO.008	LIVE	2778	474
	0406 UT.007	LIVE	3562	551
	0406 US.006	LIVE	5470	7.07

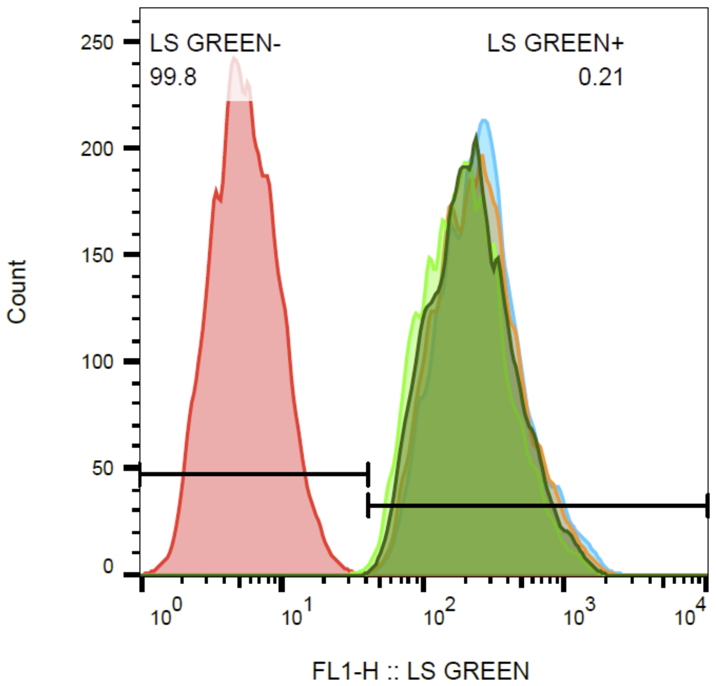

	Sample Name	Subset Name	Count	Geometric Mean : FL1-H
	0601 MPAB-.005	LIVE	9507	220
	0601 MPAB+.004	LIVE	9489	197
	0601 DMSO.003	LIVE	9426	241
	0601 UT.002	LIVE	9514	256
	0601 US.001	LIVE	9459	5.67

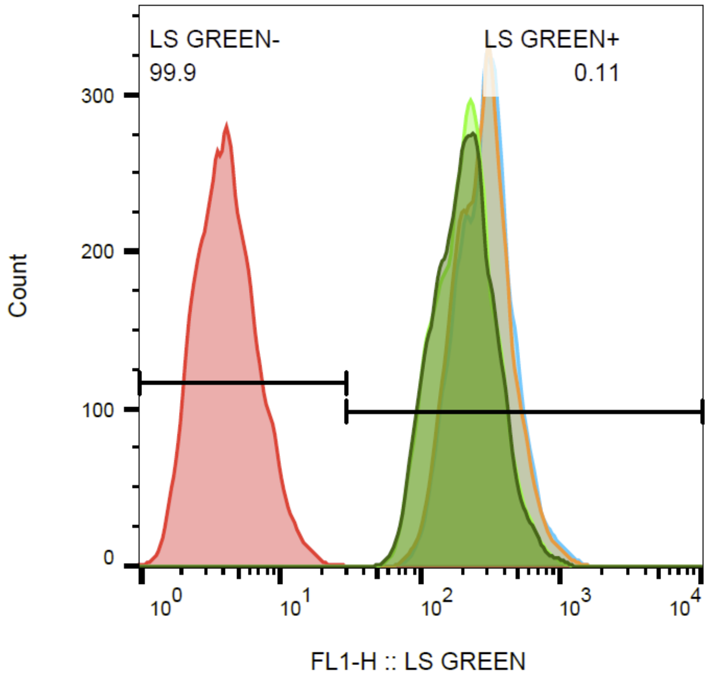

	Sample Name	Subset Name	Count	Geometric Mean : FL1-H
	HT0817 MPAB -.005	LIVE	9801	207
	HT0817 MPAB +.004	LIVE	9827	210
	HT0817 DMSO.003	LIVE	9798	278
	HT0817 UT.002	LIVE	9843	288
	HT0817 US.001	LIVE	9688	4.25

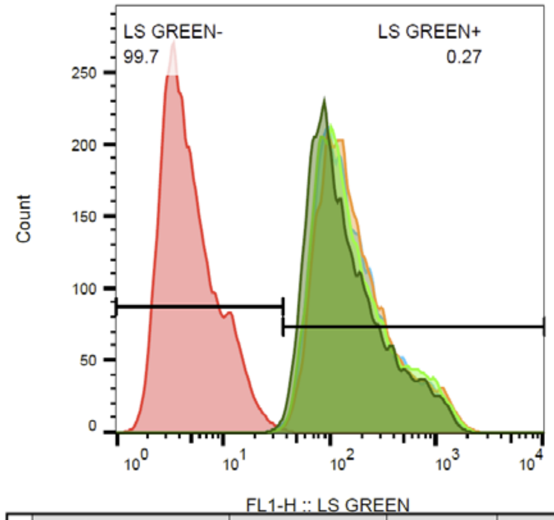

	Sample Name	Subset Name	Count	Geometric Mean : FL1-H
	HT 1025 MPAB 1NM -.005	LIVE	9672	145
	HT 1025 MPAB 1NM +.004	LIVE	9611	164
	HT 1025 DMSO.003	LIVE	9490	172
	HT 1025 UT.002	LIVE	9580	162
	HT 1025 US.001	LIVE	9329	5.03

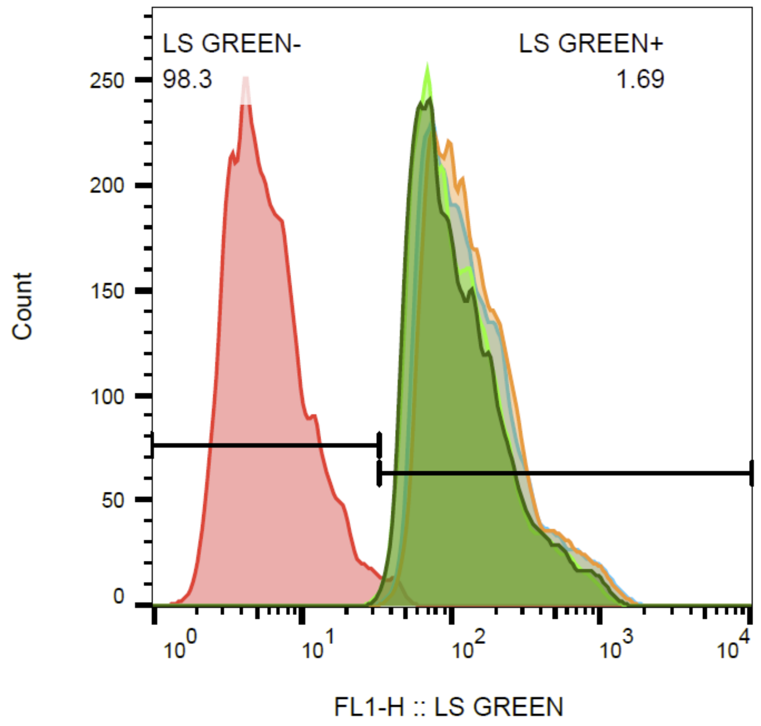

Sample Name	Subset Name	Count	Geometric Mean : FL1-H
1109 mpab 1-.005	LIVE	9718	113
1109 mpab 1+.004	LIVE	9702	113
1109 dmso.003	LIVE	9728	143
1109 ut.002	LIVE	9736	136
1109 us.001	LIVE	9650	6.15

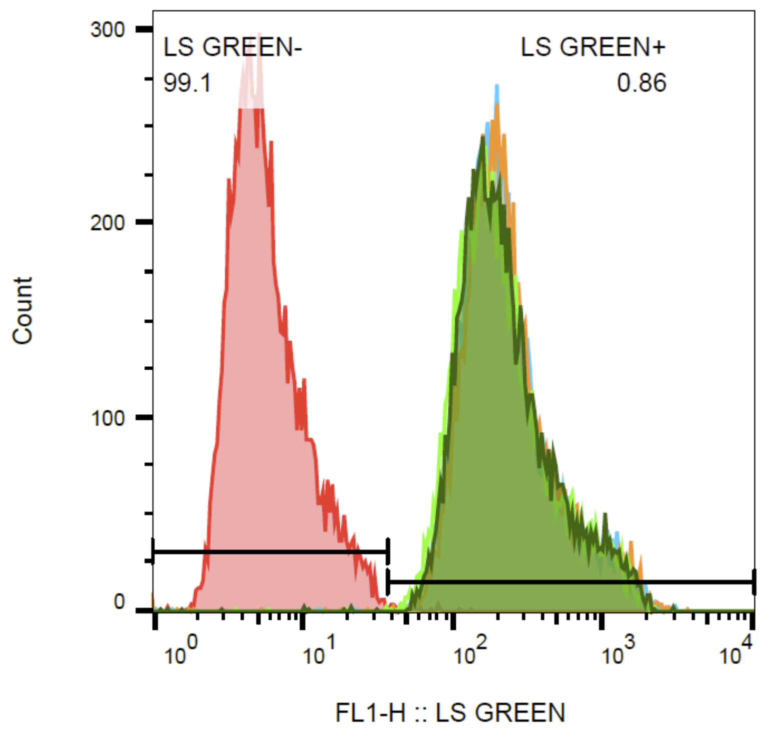

Sample Name	Subset Name	Count	Geometric Mean : FL1-H
1110 mpab 1-.010	LIVE	9547	227
1110 mpab 1+.009	LIVE	9486	214
1110 dmso.008	LIVE	9500	235
1110 ut.007	LIVE	9614	227
1110 us.006	LIVE	9512	6.04

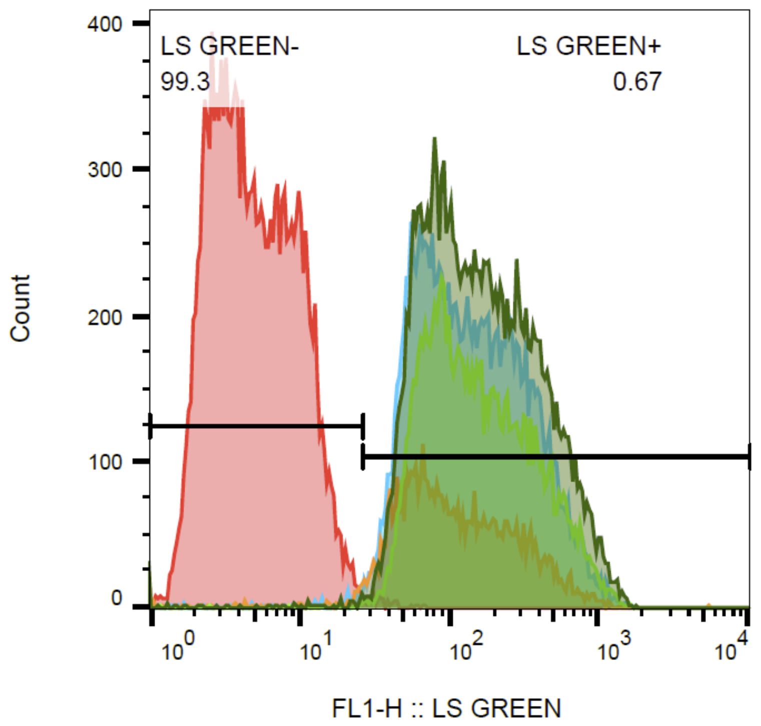

	Sample Name	Subset Name	Count	Geometric Mean : FL1-H
	1122 mpab -.004	LIVE	18351	158
	1122 mpab +.003	LIVE	11589	157
	1122 dmso.005	LIVE	5525	105
	1122 ut.002	LIVE	15502	137
	1122 us.001	LIVE	17914	5.05

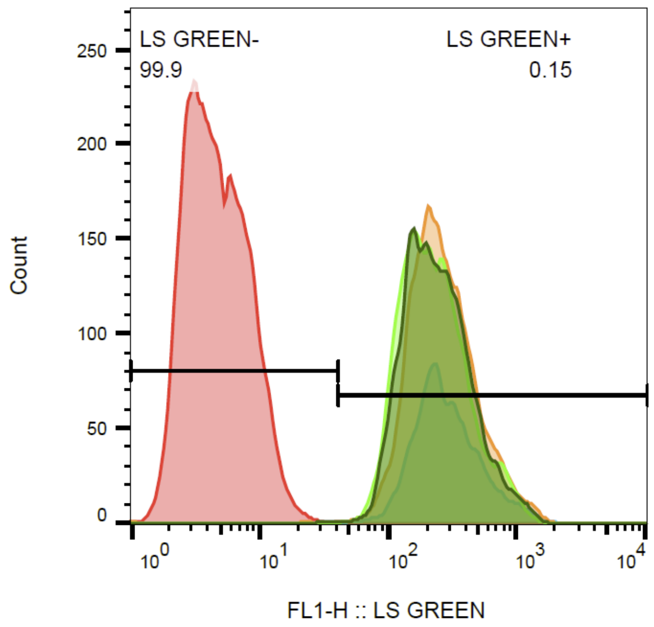

	Sample Name	Subset Name	Count	Geometric Mean : FL1-H
	1123 MPAB -.010	LIVE	6599	233
	1123 MPAB +.009	LIVE	6661	226
	1123 DMSO.008	LIVE	6502	256
	1123 UT.007	LIVE	3072	264
	1123 US.006	LIVE	9513	4.79

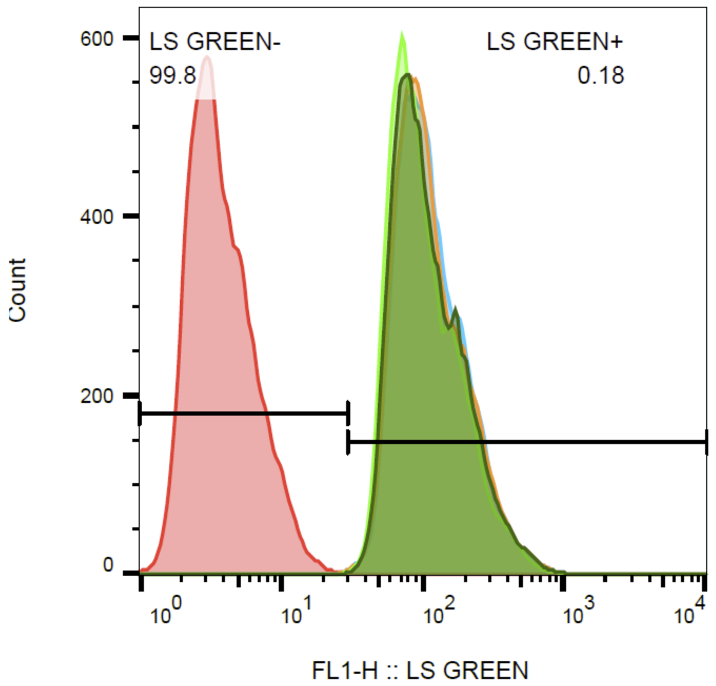

	Sample Name	Subset Name	Count	Geometric Mean : FL1-H
	1130 #1 mpab -.005	LIVE	19672	111
	1130 #1 mpab +.004	LIVE	19754	104
	1130 #1 dmso.003	LIVE	19661	114
	1130 #1 ut.002	LIVE	19744	116
	1130 #1 us.001	LIVE	19549	3.89

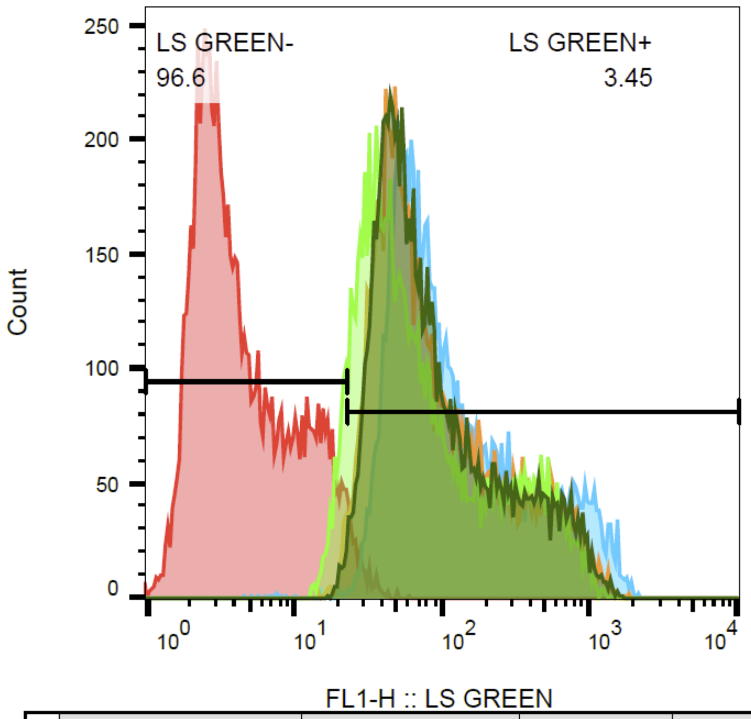

	Sample Name	Subset Name	Count	Geometric Mean : FL1-H
	HT1120#4 MPAB-.005	LIVE	9380	93.7
	HT1120#4 MPAB+.004	LIVE	9295	76.9
	HT1120#4 DMSO.003	LIVE	9267	93.9
	HT1120#4 UT.002	LIVE	9103	127
	HT1120#4 .001	LIVE	9105	4.74

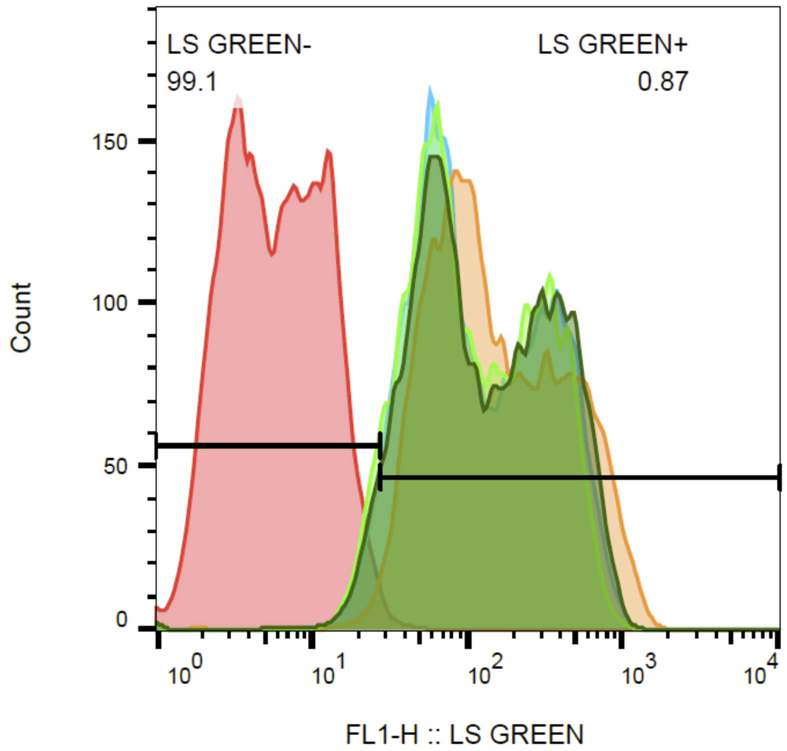

	Sample Name	Subset Name	Count	Geometric Mean : FL1-H
	HT1214#3 MPAB-.010	LIVE	9448	126
	HT1214#3 MPAB+.009	LIVE	9461	111
	HT1214#3 DMSO.008	LIVE	9150	152
	HT1214#3 UT.007	LIVE	9439	120
	HT1214#3 US.006	LIVE	9200	5.98

Original data for Figure 4

Unstaining

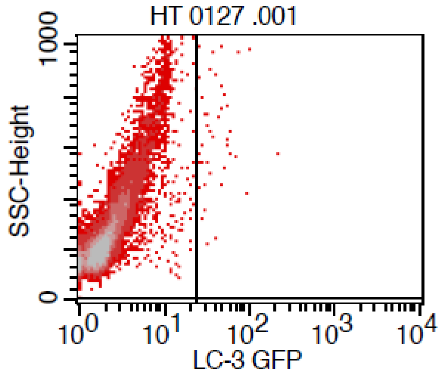

File: HT 0127 .001 Gate: G1

Quad	% Gated	% Total	X Geo Mean
UL	99.78	97.10	2.15
UR	0.22	0.21	39.92

DMSO

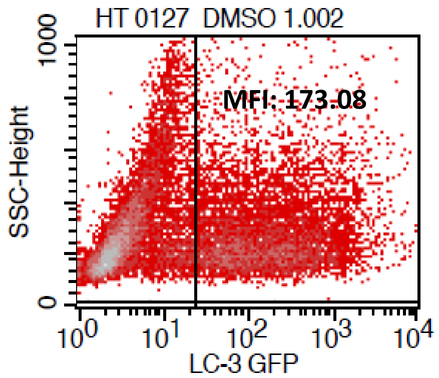

File: HT 0127 DMSO 1.002 Gate: G1

Quad	% Gated	% Total	X Geo Mean
UL	57.04	53.55	4.18
UR	42.96	40.34	173.08

MPA

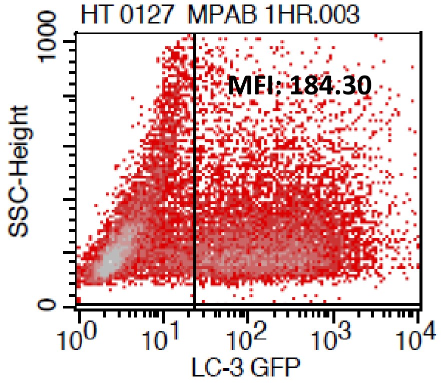

File: HT 0127 MPAB 1HR.003 Gate: [illegible]

Quad	% Gated	% Total	X Geo Mean
UL	46.72	42.82	4.96
UR	53.28	48.82	184.30

Unstaining

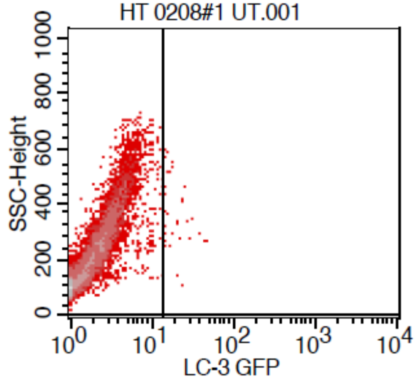

File: HT 0208#1 UT.001 Patient ID:

Quad	% Gated	% Total	X Geo Mean
UL	99.70	96.22	2.05
UR	0.30	0.29	18.86

DMSO

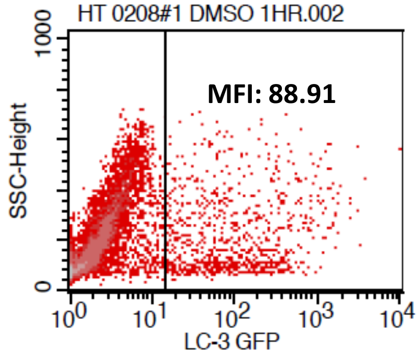

File: HT 0208#1 DMSO 1HR.002

Quad	% Gated	% Total	X Geo Mean
UL	90.45	82.81	2.15
UR	9.55	8.74	88.91

MPA

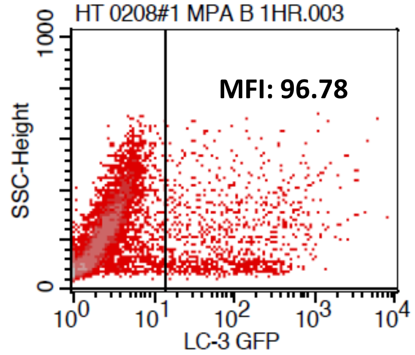

File: HT 0208#1 MPA B 1HR.003

Quad	% Gated	% Total	X Geo Mean
UL	89.59	81.59	2.21
UR	10.41	9.48	96.78

Unstaining

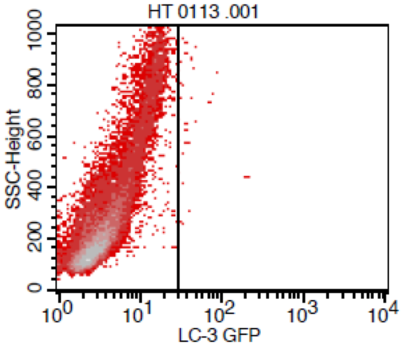

File: HT 0113 .001

Quad	% Gated	% Total	X Geo Mean
UL	99.83	91.44	3.77
UR	0.17	0.16	41.19

DMSO

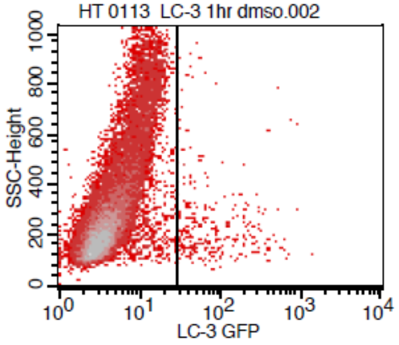

File: HT 0113 LC-3 1hr dmso.002

Quad	% Gated	% Total	X Geo Mean
UL	98.47	87.74	4.50
UR	1.53	1.36	77.92

MPA

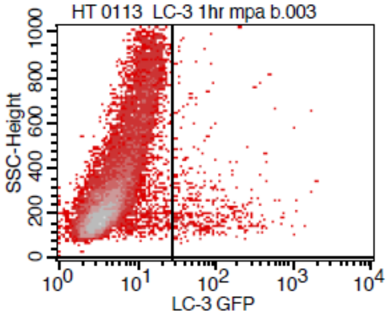

File: HT 0113 LC-3 1hr mpa b.003

Quad	% Gated	% Total	X Geo Mean
UL	97.63	85.93	5.01
UR	2.37	2.09	85.14

Unstaining

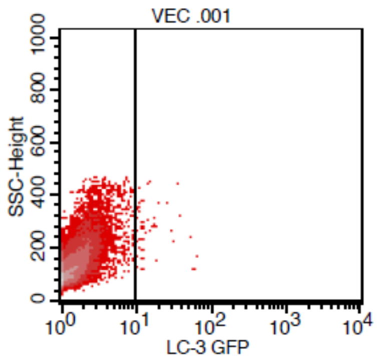

File: VEC .001

Quad	% Gated	% Total	X Geo Mean
UL	99.75	91.95	1.46
UR	0.25	0.23	15.56

DMSO

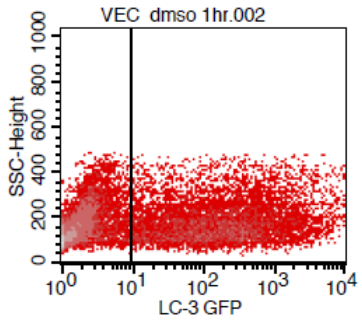

File: VEC dmso 1hr.002

Quad	% Gated	% Total	X Geo Mean
UL	53.24	49.50	1.83
UR	46.76	43.47	177.04

MPA

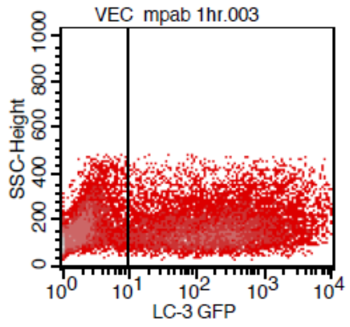

File: VEC mpab 1hr.003

Quad	% Gated	% Total	X Geo Mean
UL	40.27	37.62	2.09
UR	59.73	55.79	187.85

Unstaining

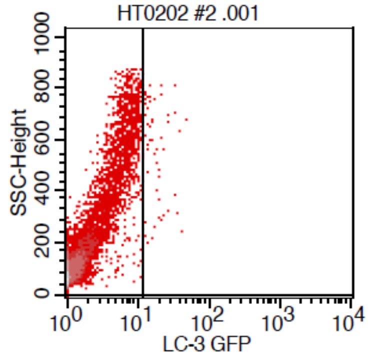

File: HT0202 #2 .001

Quad	% Gated	% Total	X Geo Mean
UL	99.14	91.90	1.60
UR	0.86	0.80	15.68
[illegible]	[illegible]	[illegible]	[illegible]

DMSO

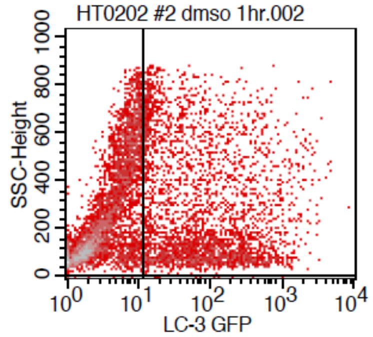

File: HT0202 #2 dmso 1hr.002

Quad	% Gated	% Total	X Geo Mean
UL	60.06	51.86	3.40
UR	39.93	34.48	78.16

MPA

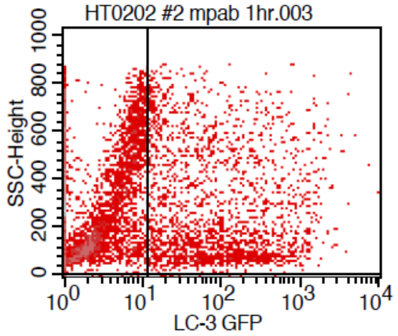

File: HT0202 #2 mpab 1hr.003

Quad	% Gated	% Total	X Geo Mean
UL	73.04	58.67	1.97
UR	26.81	21.54	81.27

Unstaining

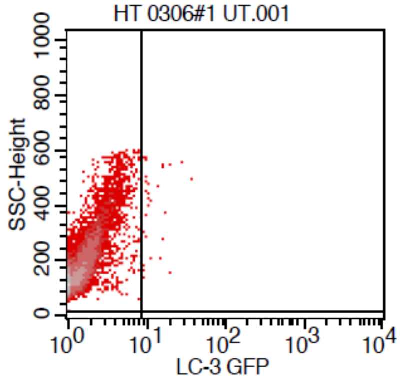

File: HT 0306#1 UT.001

Quad	% Gated	% Total	X Geo Mean
UL	99.69	92.68	1.59
UR	0.31	0.29	12.17

DMSO

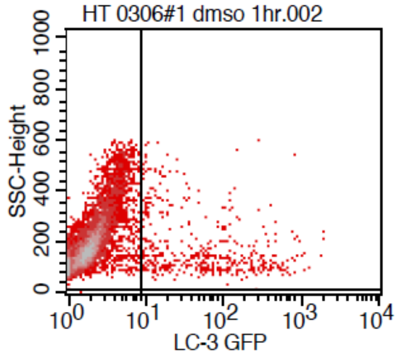

File: HT 0306#1 dmso 1hr.002

Quad	% Gated	% Total	X Geo Mean
UL	94.90	88.38	1.98
UR	5.09	4.74	52.43

MPA

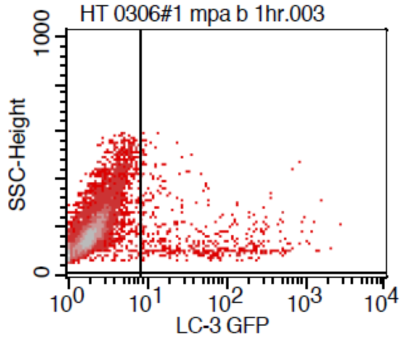

File: HT 0306#1 mpa b 1hr.003

Quad	% Gated	% Total	X Geo Mean
UL	95.18	88.71	2.04
UR	4.82	4.49	53.64

Unstaining

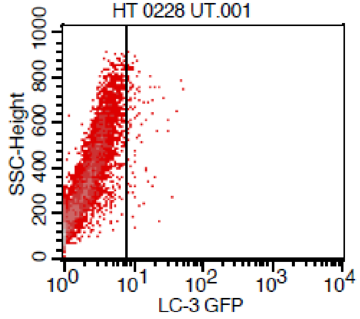

File: HT 0228 UT.001

Quad	% Gated	% Total	X Geo Mean
UL	98.66	86.97	1.91
UR	1.34	1.18	10.92

DMSO

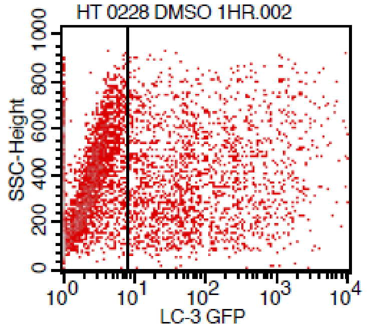

File: HT 0228 DMSO 1HR.002

Quad	% Gated	% Total	X Geo Mean
UL	66.69	50.18	1.93
UR	33.31	25.06	86.32

MPA

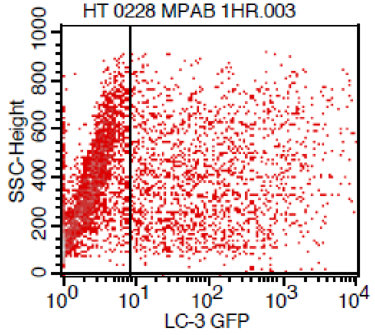

File: HT 0228 MPAB 1HR.003

Quad	% Gated	% Total	X Geo Mean
UL	63.27	45.87	2.21
UR	36.73	26.62	102.16
